# Natural lithium isotope variations in serum after lithium administration as a novel biomarker for differentiating schizophrenia and bipolar disorder

**DOI:** 10.1038/s41398-025-03627-6

**Published:** 2025-10-07

**Authors:** Junhang Dong, Baoliang Zhong, Jun Yao, Jing Chen, Shuyang Li, Pengju Xing, Xiaoliang Zhou, Gang Wang, Lvyu Yang, Ying Guo, Chengyu Hu, Zhijuan Duan, Nicholas Stanley Belshaw, Hongtao Zheng, Xing Liu, Zhenli Zhu

**Affiliations:** 1 State Key Laboratory of Geomicrobiology and Environmental Changes, School of Earth Sciences, China University of Geosciences, Wuhan, China; 2 Faculty of Material Science and Chemistry, China University of Geosciences, Wuhan, China; 3 Department of Psychiatry, Wuhan Mental Health Center, Wuhan, Hubei province China; 4 Department of Psychiatry, Affiliated Wuhan Mental Health Center, Tongji Medical College of Huazhong University of Science & Technology, Wuhan, Hubei Province China; 5 State Key Laboratory of Membrane Biology, Tsinghua-Peking Center for Life Sciences, IDG/McGovern Institute for Brain Research, School of Life Sciences, Tsinghua University, Beijing, China; 6 School of Computer Science, China University of Geosciences, Wuhan, China; 7 Retired, Department of Earth Sciences, Oxford University, Oxford, UK; 8 Hubei Key Laboratory of Yangtze Catchment Environmental Aquatic Science, Wuhan, China

**Keywords:** Physiology, Schizophrenia, Diagnostic markers, Diagnostic markers, Bipolar disorder

## Abstract

Accurate differentiation of schizophrenia (SZ) and bipolar disorder (BD) is crucial for effective clinical management. However, current diagnostic methods, which rely heavily on subjective assessments, are prone to high rates of misdiagnosis. This study pioneers the investigation of natural variations in lithium (Li) isotopes as potential biomarkers for differentiating BD and SZ. We identified significant and distinct variations in the isotopic compositions of Li in serum (δ^7^Li_serum_) of SZ patients relative to BD patients and health controls. Furthermore, we established a machine learning model that achieved a remarkable 100% accuracy in distinguishing between SZ and BD patients based on δ^7^Li_serum_ fingerprints and concentrations of biologically relevant elements (Ca, Mg, Zn, and Se) in serum. Our research reveals that δ^7^Li_serum_ is notably lighter in both BD and SZ patients (approximately 11 and 5‰, respectively) compared to that of the ingested Li drugs and decreases over time, primarily due to renal excretion. Additionally, in induced pluripotent stem cell (iPSC) models, we observed substantially heavier intracellular δ^7^Li values (up to 10‰) compared to the culture medium (0‰), likely originating from specific intracellular biochemical processes associated with competitions between Li^+^ and Mg^2+^. These differences in intracellular processes may significantly contribute to the observed distinctions in δ^7^Li_serum_ values between BD and SZ patients. Our findings demonstrate that the δ^7^Li_serum_ fingerprints in homeostasis provide valuable insights into the differentiate biomarker and pathological mechanism research of mental diseases.

## Introduction

Schizophrenia (SZ) and bipolar disorder (BD) are severe psychiatric disorders that impose a significant societal and familial burden [1–3]. Accurate diagnosis of SZ and BD is crucial for appropriate and effective treatment. However, although current diagnoses follow structured DSM-5 and ICD-10 criteria, they ultimately rely on clinician’s interpretation of subjective patient-reported symptoms and behavioral manifestations [4, 5]. This frequently experiences a high rate of misdiagnosis between SZ and BD patients due to the similarity of symptoms [6]. Numerous studies have attempted to develop diagnostic indicators through genetics, epigenetics, and metabolic characteristics, though the findings from these studies remain controversial [7–9]. Therefore, it is of great significance to develop an objective, pathogenetically related, and clinically accessible biomarker to accurately differentiate between SZ and BD.

Biologically relevant metal elements (BREs) are widely involved in biochemical processes of homeostasis, their abnormal metabolisms are often inextricably associated with the occurrence of diseases [10–12]. Previous studies have identified disruptions in the homeostasis of BREs in patients with SZ and BD [13, 14]. However, the differences in the variations of BREs concentrations in body fluids, stemming from the distinct pathologies of these two disorders, lack sufficient sensitivity to clearly distinguish SZ and BD [15]. Notably, natural stable isotopic fractionation, a powerful tool widely used in earth science [16, 17], has gained considerable attention in medical research [18–21]. The isotopic composition of BREs in body fluids shows significant changes associated with abnormal metabolisms [22–24], demonstrating higher sensitivity than its concentration variations and offering potential as diagnostic/prognostic tools [25–27]. However, since these BREs are continuously ingested, any abnormal isotopic fractionations caused by diseases in homeostasis potentially masked by dietary factors [28]. Additionally, medication containing metal salts are another significant source of metal elements in homeostasis, offering a novel perspective independent of diet. As far as we can discern, no studies have been conducted on the isotopic fractionation of metal elements originating from metallodrugs.

Lithium (Li) medication is widely recognized as the gold standard mood-stabilizing agent for BD [29]. In addition, it has also shown anti-suicide effects for SZ patients, and even within the general population [1]. However, it should be noted that the therapeutic effect of Li on typical symptoms of BD is significantly greater than that of SZ, which can be attributed to variations in the Li^+^ therapeutic targets, such as glycogen synthase kinase-3β (GSK-3β) [30]. Li has two naturally occurring isotopes, namely ^7^Li (92.41%) and ^6^Li (7.59%) [31]. The fractionation of Li isotopes in marine carbonates has been extensively used as a powerful proxy to trace global weathering, considering Li’s biological inertness from a geological perspective [16, 32–34]. However, several studies have shown clear differences in Li concentrations and natural variations in its isotopic composition (δ^7^Li) among various organs in mammals and marine organisms [35–37]. These studies support the existence of significant Li isotopic fractionation in various biochemical processes in organisms. More importantly, theoretical studies on the molecular therapeutic mechanism of Li^+^ have demonstrated that Li^+^ selectively compete with Mg^2+^-binding sites of specific enzymes associated with typical BD symptoms, such as GSK-3β [38–40]. These selective bonding-driven competition processes are potentially accompanied by Li isotopic fractionation, providing the potential to develop novel biomarkers for distinguishing between BD and SZ.

In this study, we conducted the first investigation into the natural variations of the Li isotopic compositions in serum (δ^7^Li_serum_) obtained from patients with BD, SZ, and health controls (HC) undergoing Li treatment. We found significant differences in δ^7^Li_serum_ between BD and SZ patients, and developed a classification model for distinguishing between BD and SZ using a random forest (RF) machine learning algorithm based on the δ^7^Li_serum_ values and several BREs concentrations (Ca, Mg, Zn, and Se) in serum. To gain a deeper understanding of the causes of the δ^7^Li_serum_ variations and the disparities in δ^7^Li_serum_ between BD and SZ, we further explored the impact of urinary excretion, transport, and intracellular processes on δ^7^Li_serum_ variation. Additional experiments, conducted using induced pluripotent stem cell (iPSC) models, suggested that intracellular Li isotopic fractionation associated with Li^+^’s competition with Mg^2+^ likely contributes to the observed difference in δ^7^Li_serum_ values between BD and SZ. Our work underscores the significance of biological Li isotopic fractionation and offers novel insights into intracellular Li therapeutic mechanism and psychiatric pathology from an isotopic perspective.

## Methods

### Samples harvesting

This study included a total of 53 BD patients (male: female = 15: 38; age: 27.7 ± 13.2), 30 SZ patients (male: female = 24: 6; age: 47.7 ± 9.2), and 14 HC subjects (male: female = 6: 8; age: 24.4 ± 1.8) undergoing Li medication. All blood and urine samples from HC subjects and patients with BD/SZ were collected from Wuhan Mental Health Center between 2021 and 2023, and signed informed consents were obtained from participants or their authorized representatives prior to all study procedures. Blood samples were drawn approximately 12 h after the last Li dose and collected in serum separator tubes to obtain serums. Participants of BD/SZ patients with cancer or other diseases, as well as those who took Li medication for fewer than 7 days, were excluded. To ensure comparability δ^7^Li_serum_ values between groups, 14 HC participants were administered Li medication for more than 7 days under clinical supervision. All patients with BD and SZ were inpatients at Wuhan Mental Health Center, with confirmed diagnoses made at least three months prior to sample collection. Their symptoms had been effectively stabilized through ongoing medical treatment. Although BD and SZ are spectrum disorders with varying degrees of severity, the inclusion of clinically stable, hospitalized patients helped minimize individual variability in this study. To obtain both serum and urine samples at 6 and 12 h post-dose, we invited patients to voluntarily participate in an additional time-resolved sampling protocol. Seven BD patients consented to this protocol and provided paired serum and urine samples at both time intervals. Supplementary Table 1–3 summarizes the clinical characteristics of the study participants. To better explain the observed δ^7^Li_serum_ differences between BD and SZ groups, we also investigated intracellular Li isotopic fractionation behavior using laboratory-cultured iPSCs derived from BD patients and HC subjects, which were cultured in media containing Li^+^ at natural physiological concentrations. SZ patient-derived iPSCs were not incorporated in this study owing to current technical constraints and limited availability. Details regarding reagents, cell culture procedures and experimental workflow are provided in the Supplementary Materials and Figs. S1–3. The authors assert that this study was conducted in accordance with the principles of the Declaration of Helsinki, and the study protocol was approved by the ethical committees of China University of Geosciences (Wuhan) and Wuhan Mental Health Center. The sample size was sufficient to support all planned analyses. All investigators involved in data measurements and analysis were blinded to group assignments until all measurements were completed and the dataset was locked for analysis.

### Analysis of δ^7^Li values and BREs concentrations

The δ^7^Li values were measured using a multiple collector inductively coupled plasma mass spectrometer (MC-ICP-MS) (Nu Plasma II, Nu instruments, UK) at the State Key Laboratory of Geomicrobiology and Environmental Changes (GMEC), China University of Geosciences, Wuhan. The BREs concentration were analyzed using an inductively coupled plasma quadrupole mass spectrometer (ICP-QMS) (7900, Agilent). The details of pretreatment process and δ^7^Li measurement processes are listed in the Supplementary Materials and Figs. S4–5.

### Machine learning

The machine learning was performed in PyCharm. The packages from scikit-learn was used for classification, including random forest (RF), neural network (NN), and decision tree (DT). Assessment of the relative importance of variables was included within the RF model. Normalization was performed separately for datasets and calculated as: (x−min)/(max−min). The RF classifier was trained to predict disease types, with the output being the predicted classes.

### Statistical analysis

Data are presented as mean ± SD for the BREs and Li concentrations, and as mean ± 2 SD for the δ^7^Li results. The normality of continuous variables was assessed using the Shapiro-Wilk test, while the homogeneity of variances was evaluated with Levene’s test. Depending on data characteristics, a students’ t-test was used to compare the BREs concentrations and δ^7^Li_serum_ values in serum of the different groups. A Pearson correlation test was used as appropriate. Statistical significance is defined by *P*-values < 0.05 (denoted by an asterisk, *), with more asterisks corresponding to ever-higher levels of significance (**P ≤ 0.01 and ***P ≤ 0.001). All calculations were performed using SPSS (version 23.0).

## Results

### Significant but insufficient difference of BREs concentrations in serum between BD and SZ

**Fig. 1:**
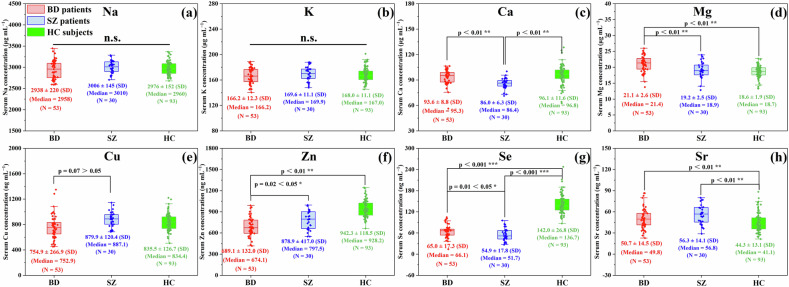
Significant but insufficient difference of BREs concentrations in serum between BD and SZ. The (**a**) Na, (**b**) K, (**c**) Ca, (**d**) Mg, (**e**) Cu, (**f**) Zn, (**g**) Se, and (**h**) Sr concentrations in serum of BD patients (red box, N = 53), SZ patients (blue box, N = 30) and HC subjects (green box, N = 93). The data are presented as box-and-whiskers graphs, with the box extending from the 25th–75th percentiles, the horizontal line representing the median.

As essential components of enzymes and hormones, BREs play key roles in various biological systems. Previous studies have reported significant differences in serum concentrations of BREs amongst BD, SZ and HC subjects [15, 41]. In this study, we firstly evaluated the concentrations of several widely recognized BREs in serum and investigated their potential to differentiate between BD and SZ. As illustrated in Fig. 1a–h, there are no significant differences in serum Na and K concentrations among the BD, SZ, and HC groups. However, we observed significant differences in serum Ca (*P* < 0.01), Sr (*P* < 0.01), and Se (*P* < 0.001) concentrations between SZ patients and HC subjects. Additionally, significant differences were found in serum Mg (*P* < 0.01), Zn (*P* < 0.01), Sr (*P* < 0.01), and Se (*P* < 0.001) concentrations between BD patients and HC subjects. Furthermore, significant differences were evident in serum Ca (*P* < 0.01), Mg (*P* < 0.01), Zn (*P* < 0.05), and Se (*P* < 0.05) concentrations between BD and SZ groups. The serum Cu concentrations did not show significant differences among these three groups. However, the difference in serum Cu between the BD and SZ groups (*P* = 0.07) was close to the significance threshold of 0.05, which warranted its inclusion in the subsequent receiver-operating characteristic (ROC) curve analysis. The ROC curves depicted in Fig. S6 show that the area under the ROC curve (AUC) reached 0.660, 0.707, 0.867, 0.998, 0.682, and 0.555 for Ca, Mg, Zn, Se, Sr, and Cu, respectively, demonstrating serum Se concentration is the most effective in distinguishing patients with either BD or SZ from HC subjects. However, the discriminating effect between BD and SZ was not sufficient, as evidenced by AUC values of only 0.792, 0.720, 0.705, and 0.675 (all below 0.8) for Ca, Mg, Zn, and Se, respectively (Fig. S7), showing that the variations of serum BREs concentrations are insufficient to differentiate SZ and BD patients.

### Distinct δ^7^Li_serum_ signatures between BD and SZ patients

**Fig. 2:**
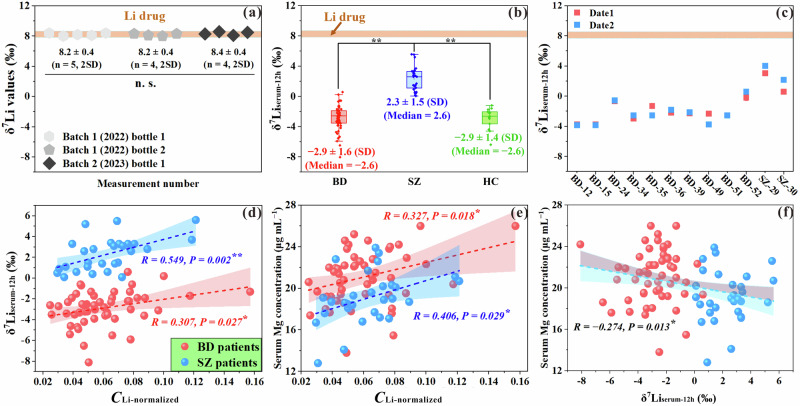
Variations in lithium isotopes in serums of BD and SZ patients relative to ingested Li drugs. **a** The δ^7^Li values of Enhua Li drugs with different production batches and bottles. Batch 1 and Batch 2 represent two batches of Enhua Li drugs produced in 2022 and 2023, respectively. **b** The comparison of the δ^7^Li_serum-12h_ between BD patients (red box, N = 53), SZ patients (blue box, N = 30), and HC subjects (green box, N = 14). The data are presented as box-and-whiskers graphs, with the box extending from the 25th–75th percentiles, and the horizontal line representing the median. **c** Comparison of δ^7^Li_serum-12h_ in patients with BD (N = 10) and SZ (N = 2) between serum samples collected on two different dates. **d** The correlation between the δ^7^Li_serum-12h_ and (**e**) serum Mg concentration with the *C*_Li-normalized_ (ranging from 0.02–0.16). **f** The correlation between the serum Mg concentration and the δ^7^Li_serum-12h_. The red plots and blue plots of (**d**), (**e**), and (**f**) represent patients with BD and SZ, respectively.

Our study consistently used Enhua Li medication to ensure uniformity in the δ^7^Li values of Li drugs (δ^7^Li_drug_), thereby eliminating the variations in initial δ^7^Li levels among patients that could arise from different Li drug brands (Supplementary Materials and Fig. S8). We compared the δ^7^Li_drug_ values of different Li tablets (Enhua) from two distinct bottles of the same batch and from one bottle of a different production batch. No significant difference was observed among these tablets, with an average δ^7^Li_drugs_ value of +8.3 ± 0.4‰ (Fig. 2a). This information allows us with confidence to investigate the differences in δ^7^Li_serum_ attributable to distinct metabolic pathways and pathogenesis among patients with BD and SZ.

Subsequently, the δ^7^Li values in serum samples collected 12 h after taking the Li drug (δ^7^Li_serum-12h_) were measured. No significant difference was found between the patients with BD and SZ. Relative to the initial δ^7^Li_drugs_ (+8.3 ± 0.4‰), the δ^7^Li_serum-12h_ values exhibited a clear fractionation in favor of lighter ^6^Li isotope for both BD (−2.9 ± 1.6‰, N = 53) and SZ ( + 2.3 ± 1.5‰, N = 30) patients (Fig. 2b) in serum. Meanwhile, these is a clear difference (P^***^ < 0.001) between means of δ^7^Li_serum-12h_ for these two groups (exceeding 5‰), which is much larger than the second-order variations within each group (~1.5‰). Additionally, the day-to-day variabilities of δ^7^Li_serum-12h_ were evaluated, revealing a conserved and reproducible fingerprint with differences within 1‰ among most patients between two sampling dates (Fig. 2c). In the absence of any correlation between δ^7^Li_serum-12h_ and anthropometrical parameters (Figs. S9–12), we conducted additional HC experiments with a small group of young volunteers (N = 14) with the same level of Li medication as a further control study (Table S3). The results revealed that the δ^7^Li_serum-12h_ values of HC subjects were similar to those of BD patients (*P* > 0.05), while significantly lighter than those of SZ patients (*P* < 0.01, Fig. 2b).

A moderate correlation exists between normalized serum Li levels (*C*_Li-normalized_), defined as the serum Li concentration (mg kg^−1^) divided by the Li dosage per unit body weight (mg kg^−1^), and the δ^7^Li_serum-12h_, with *R*-values of 0.289 (*P* = 0.04) and 0.549 (*P* = 0.002) for patients with BD and SZ, respectively (Fig. 2d). Moreover, a moderate positive and negative correlation was found between serum Mg concentration with *C*_Li-normalized_ (Fig. 2e) and δ^7^Li_serum-12h_ (Fig. 2f), respectively, in both BD and SZ patients.

### Preferential urinary excretion of heavier ^7^Li isotopes

**Fig. 3:**
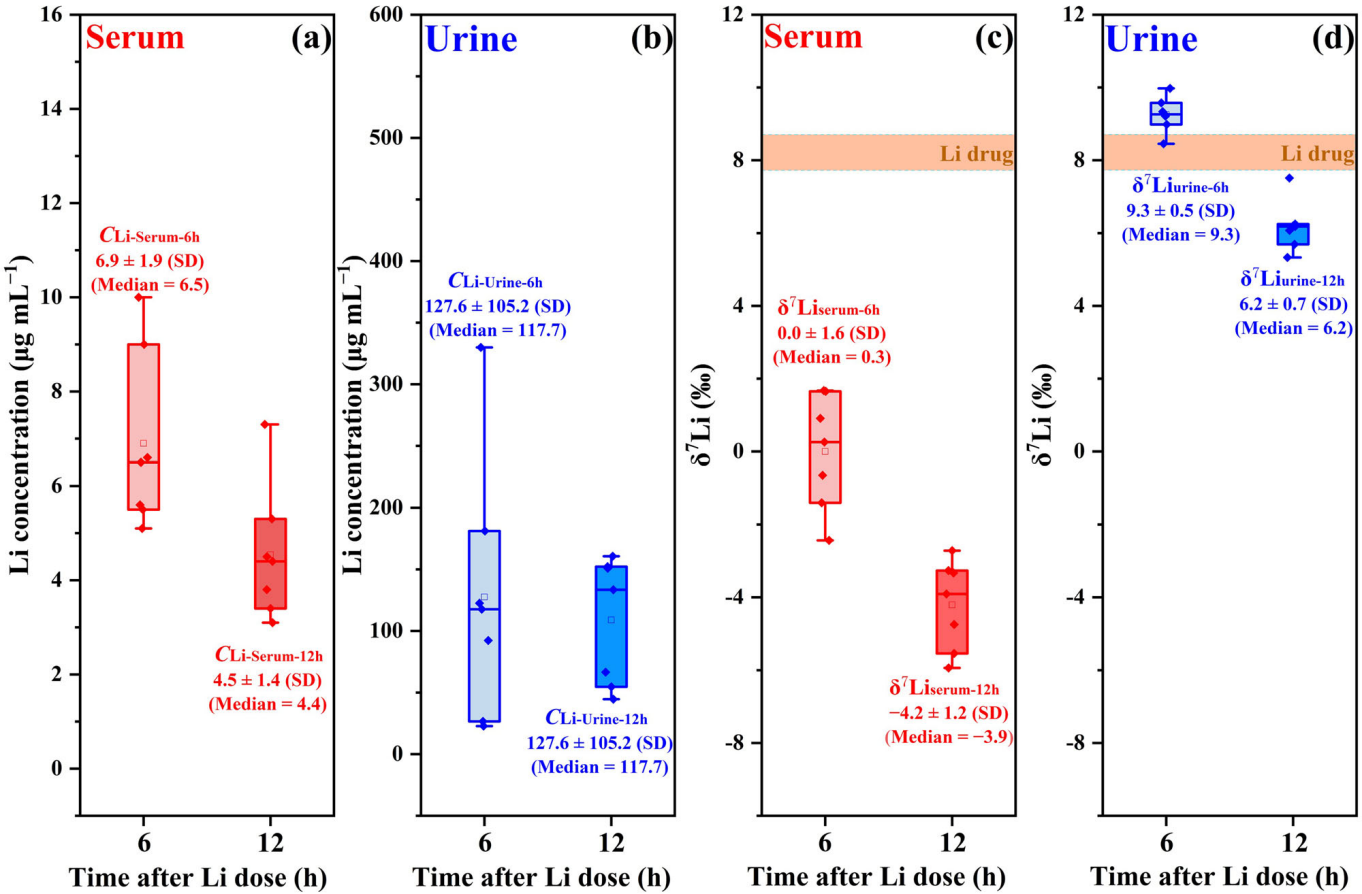
Time-dependent changes of Li concentration and δ^7^Li in serum and urine following medication. Panels show the variations of Li concentration in (**a**) serum (red boxes, *C*_Li-serum-6h_ and *C*_Li-serum-12h_) and (**b**) urine (blue boxes, *C*_Li-urine-6h_ and *C*_Li-urine-12h_), as well as corresponding variations of δ^7^Li values in (**c**) serum (red boxes, δ^7^Li_serum-6h_ and δ^7^Li_serum-12h_) and (**d**) urine (blue boxes, δ^7^Li_urine-6h_ and δ^7^Li_urine-12h_) from seven individuals after 6 h and 12 h of Li_2_CO_3_ dosage.

To better understand the variation observed in δ^7^Li_serum-12h_ between patients with BD and SZ, it is necessary to consider the Li metabolic pathway that may affect δ^7^Li_serum_ values. Unfortunately, there is currently a limited understanding of Li isotopic fractionation in biological processes [35, 37, 42]. The positive correlations observed between δ^7^Li_serum-12h_ and *C*_Li-normalized_ in both BD and SZ patients indicate that the loss of Li from homeostasis may lead to a decrease in the δ^7^Li_serum-12h_. Urination is the primary excretion process for ingested Li and consequently may affect δ^7^Li_serum-12h_ [43], but currently, no studies have examined the Li isotopic fractionation associated with this process. To address this lack of information, we measured the Li concentrations and δ^7^Li values in both serum and urine samples collected from seven BD patients, specifically at two distinct time intervals fixed at 6 and 12 h after Li intake. As illustrated in Fig. 3a, b, the serum Li concentrations exhibited general decreasing trend from 6 h (6.9 ± 1.9 μg mL^−1^) to 12 h (4.5 ± 1.4 μg mL^−1^) after Li dose, whereas the urine Li concentrations remained relatively stable, changing from 127.6 ± 105.2 to 109.0 ± 51.3 μg mL^−1^. Notably, both the δ^7^Li_serum_ (from 0 ± 1.6 to −4.2 ± 1.2‰, Fig. 3c) and δ^7^Li_urine_ (from 9.3 ± 0.5 to 6.2 ± 0.7‰, Fig. 3d) exhibited a notable decrease from 6 to 12 h. Importantly, the mean δ^7^Li_urine_ consistently showed a heavier value, approximately 10‰, higher than the mean δ^7^Li_serum_ at both distinct time intervals, with δ^7^Li_urine-6h_ values even heavier than δ^7^Li_drugs_ values.

### Significant enrichment of heavier ^7^Li isotopes in cells exposed to natural Li concentrations

**Fig. 4:**
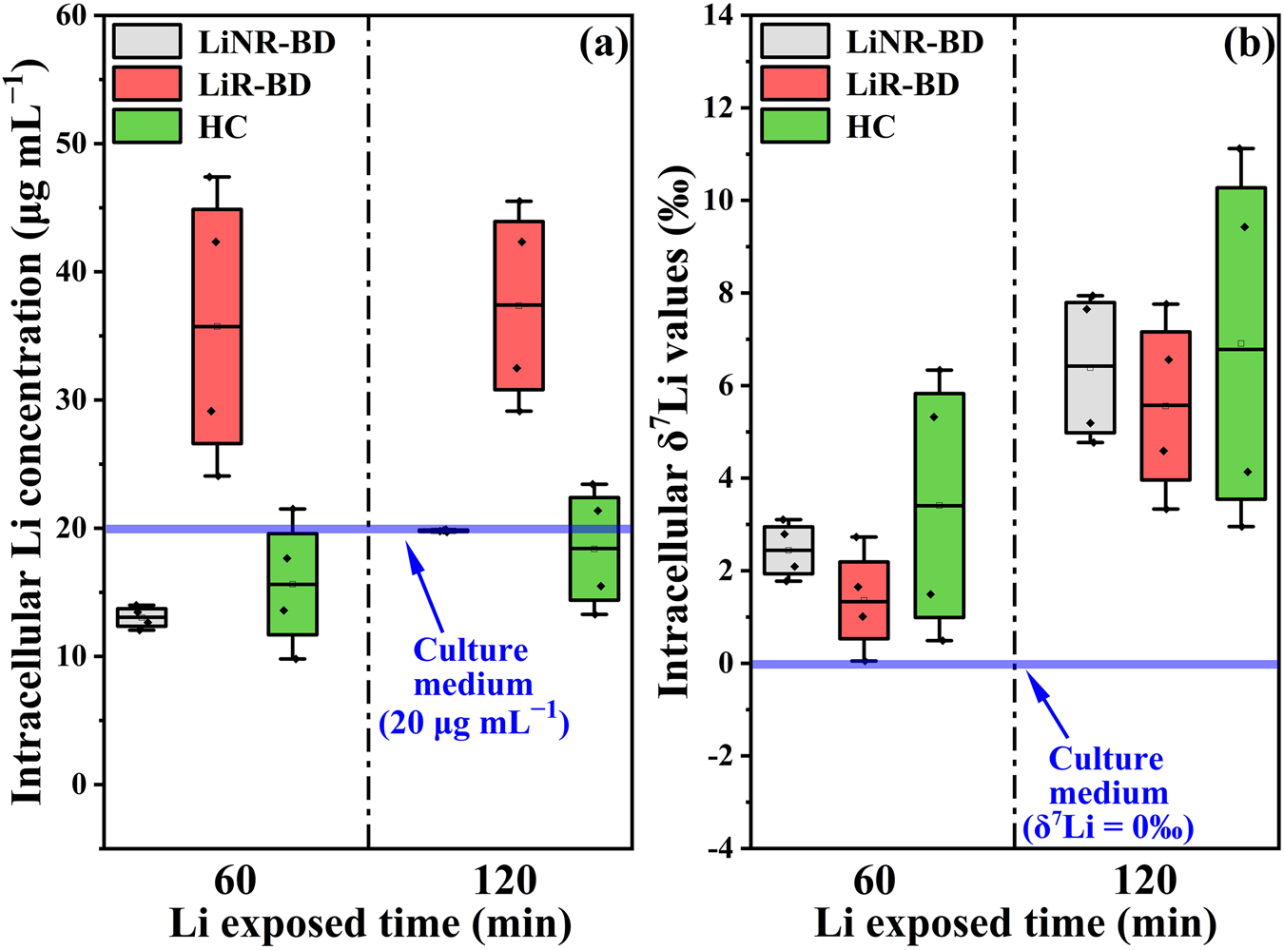
Characterization of Li isotopic fractionation during intracellular processes. The intracellular (**a**) Li concentrations and (**b**) δ^7^Li values of iPSCs from LiR-BD (N = 4 from 2 cell lines), LiNR-BD (N = 4 from 2 cell lines), and HC subjects (N = 4 from 2 cell lines) at Li expose time of 60 and 120 min.

Clearly, investigation of intracellular Li isotopic fractionation processes is of great importance. Previous studies by Poet et al. [42] pioneered investigations into Li isotopic fractionation during the transmembrane transport process using fibroblasts cell models. Their study conducted at external Li concentrations well above natural levels (over 100 μg mL^−1^) shows that membrane ion channels and Na^+^-Li^+^/H^+^ exchangers (NHEs) fractionate Li isotopes, with preferential uptake of lighter ^6^Li isotopes during transmembrane transport. However, this finding does not account for the heavier δ^7^Li values observed in specific tissues (Fig. S13), prompting further inquiry into intracellular biological processes contributing to the accumulation of heavy ^7^Li isotopes. To directly address this question, we investigated cellular δ^7^Li signatures using in vitro iPSC lines derived from two Li-responsive BD patients (LiR-BD), two Li non-responsive BD patients (LiNR-BD), and two HC subjects, with extracellular Li concentrations of 20 μg mL^−1^ over a period of 120 min. The data presented in Fig. 4a shows that the intracellular Li concentration in LiR-BD iPSCs was nearly twice as high as those of iPSCs from LiNR-BD and HC subjects. These measured intracellular Li concentrations all approached nearly maximum values after an exposure time of 60 min (Fig. S14). Moreover, the intracellular Li concentration exhibited minimal changes, with LiR-BD cells showing no significant variations (from 35.7 ± 11.7 to 37.3 ± 8.2 μg mL^−1^) and only moderate increases for LiNR-BD cells (from 13.1 ± 0.9 to 19.8 ± 0.1 μg mL^−1^) and HC cells (from 15.6 ± 5.9 to 18.4 ± 5.1 μg mL^−1^) as the exposure time to Li increased from 60 to 120 min (Fig. 4a). Interestingly, the intracellular δ^7^Li values of all six iPSC lines were approximately 2.4‰ heavier (ranging from 1.3 to 3.4‰) than the culture medium (δ^7^Li = 0‰) at 60 min. This enrichment further increased to approximately 6.5‰ (ranging from 5.6 to 7.0‰) at 120 min (Fig. 4b), indicating a significant cellular enrichment of the heavier ^7^Li isotope relative to the culture medium.

### Data modeling

**Fig. 5:**
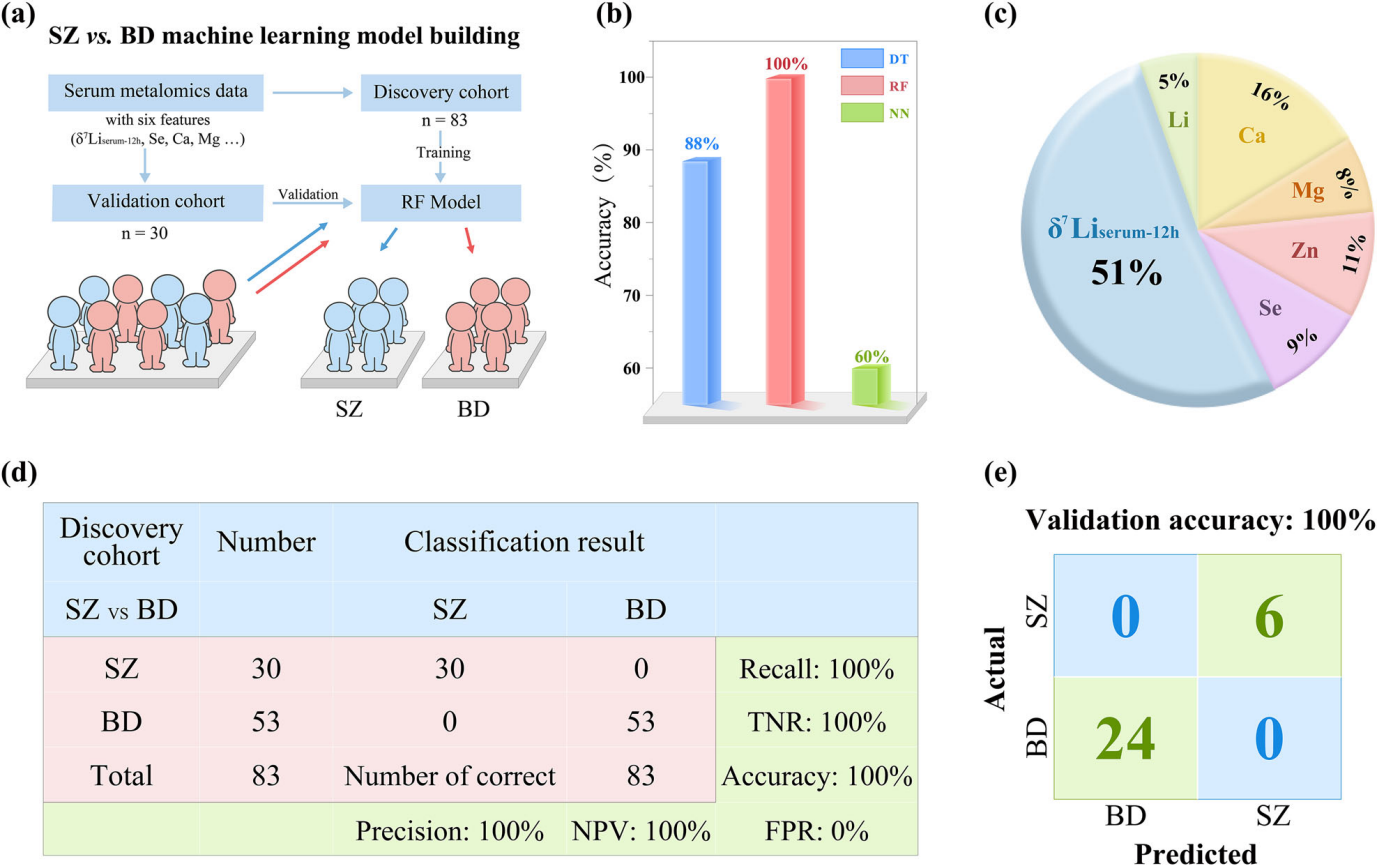
Data modeling. **a** Schematic of the machine learning model for classification of BD and SZ. **b** Accuracy and sensitivity evaluation of three machine learning algorithms (NN, RF, and DT) based on 6 features, including δ^7^Li_serum-12h_, *C*_Li-normalized_, serum Se, serum Ca, serum Mg, and serum Zn. **c** The variable importance of variables in the RF model. **d** The classification results and model performance. The “number of correct” means the number of subjects with correct classification results. NPV negative predictive value, TNR true negative rate, FPR false positive rate. **e** the practical accuracy of the machine learning model for validation cohort.

Examination of the acquired data suggests significant potential for utilizing δ^7^Li_serum-12h_ to distinguish between BD and SZ patients, as evidenced by a high AUC value of 0.995 (Fig. S7b). To further enhance the differentiation between BD and SZ, a machine learning model was developed by incorporating δ^7^Li_serum-12h_, *C*_Li-normalized_, and the concentrations of four BREs (Ca, Mg, Zn, and Se) in serum (Fig. 5a). Three different algorithms, random forest (RF), neural network (NN), and decision tree (DT) were evaluated. The DT algorithm builds a flowchart-like structure, splitting data at decision nodes based on specific variable thresholds. While DT allows for straightforward interpretation, it is prone to overfitting, particularly in small datasets [44]. The NN algorithm, inspired by the structure of the human brain, consists of interconnected layers of neurons that learn complex nonlinear relationships between input features and output classes. However, NN generally require large datasets and may suffer from reduced performance when sample sizes are limited [45]. As an ensemble supervised learning method, RF constructs numerous individual decision tree classifiers on sub-samples of the entire dataset, effectively mitigating the risks of overfitting compared to single decision trees. This makes RF particularly effective for small datasets (as in our study), among these three methods, the RF exhibited the best performance with highest accuracy for the discovery cohort at 100% (Fig. 5b) compared to DT (88%) and NN (60%). Our results showed that the RF model improved the AUC to 1.000 for distinguishing BD and SZ, compared to single dimensional information of δ^7^Li_serum-12h_ (0.995) (Fig. S7b). Assessment of the relative importance of variables included within the RF model, as summarized in Fig. 5c, revealed all variables contributed to the modeling outcome, with δ^7^Li_serum-12h_ being the most influential decisive variable. The overall classification results using the RF model are presented in Fig. 5d. The classification accuracy, precision, recall rate, and TNR, also known as specificity, all reached 100%, with 0% FPR. These results indicate the robustness of the RF model in accurately differentiating SZ patients from BD patients.

Furthermore, in order to evaluate the practical accuracy of the RF model, we applied it to a separate validation cohort comprising 24 individuals diagnosed with BD and 6 individuals diagnosed with SZ. The results, as shown in Fig. 5e, demonstrated that the RF model performed exceptionally well, achieving a remarkable accuracy rate of 100%. This result provides strong evidence for the effectiveness and practicality of the RF model based on δ^7^Li_serum-12h_ in accurately discriminating between BD and SZ.

## Discussion

Currently, the diagnosis of BD and SZ heavily relies on the subjective judgment of clinicians, leading to a high rate of misdiagnosis due to the similarity of symptoms [46, 47]. As reported by Ayano et al. [6], patients with BD were frequently misdiagnosed as having SZ (60%), while patients with SZ were also more likely to be misdiagnosed as having BD (56%). Therefore, it is crucial to develop effective objective biomarkers for psychiatrists to accurately distinguish between these two diseases, even after an initial misdiagnosis. In this study, the single-dimensional δ^7^Li_serum-12h_ showed a higher AUC value of 0.995 compared to other potential diagnostic indicators based on multi-dimensional biomarkers that have recently emerged to differentiate BD and SZ, such as plasma metabolic characteristics (AUC = 0.867) [9] and extracellular vesicle biomarkers (AUC = 0.966) [4]. Furthermore, our RF model, developed based on δ^7^Li_serum-12h_ and serum BREs concentration, achieved 100% differentiation between BD and SZ using the acquired dataset (AUC = 1.000). This model’s ability was further validated by an unknown validation cohort (Fig. 5e), giving confidence in the potential diagnostic capability of our approach.

Although the specific mechanisms remain unclear, serum BREs imbalances in patients with psychiatric disorders have been widely reported [15, 41]. The serum BREs concentrations of HC subjects in our study are consistent with previous reports [48, 49], and the differences observed between BD/SZ patients and HC subjects further support the findings in Zaks’s [12] review on elemental imbalances in psychiatric disorders. Moreover, some differences of specific serum BREs concentrations were significant between SZ and BD, such as the upregulation of serum Mg concentration in BD patients (Fig. 1b), which may be related to some abnormally active specific molecular pathways associated with psychopathological symptoms, such as neurotransmitter signaling and oxidative stress [50]. However, it is undeniable that the diagnosis of BD and SZ cannot be solely achieved based on serum BREs concentrations due to the highly overlap between these two groups.

The application of stable isotopes in geochemistry is long established, making use of the differences in the masses of isotopes where distinct behaviors during physical and chemical reactions, as well as biological processes can be observed [51, 52]. The uptake, metabolism and excretion of elements can lead to distinct and measurable natural variations in their isotopic signatures, which can be used as a cutting-edge tool in the diagnosis of diseases that affect metal metabolism. Li isotopes (^7^Li and ^6^Li) exhibit the largest relative mass difference among any metal isotope pairs, at 16.6%, with the ^6^Li reacting faster in dynamic processes (kinetic effect) [53], while the ^7^Li tends to be enriched in stronger coordination and bonding environments (thermodynamic or equilibrium effect) [33]. Abnormalities in the biological processes involving metal elements in homeostasis can cause abnormal metal isotope fractionation, which can be reflected in the variation of isotope signatures in body fluids. For example, a notable association exists between a significant downregulation of serum/blood copper isotopic compositions and an upregulation of copper metabolism in cancer cells [25]. Similarly, the significant difference of δ^7^Li_serum-12h_ values is likely associated with the Li metabolic, therapeutic and pathological differences between BD and SZ. To effectively discuss the relationship between δ^7^Li_serum-12h_ and pathological characteristics, as well as Li treatment mechanisms, it is important to have an understanding of Li isotopic fractionations in key metabolic processes in homeostasis.

Li drug is primarily absorbed through the gastrointestinal tract and is typically completely absorbed within approximately three hours [43]. This means that the influence of the absorption process Li isotopic fractionation on the variation of δ^7^Li_serum-12h_ can be considered negligible. Furthermore, in patients taking Li medication, the contribution of Li from diet is also negligible [54]. Consequently, the initial δ^7^Li signature in homeostasis can be considered consistent across all the subjects who have taken the same Li medication.

The primary pathway for Li^+^ removal from the homeostasis is through urine [43], which could affect the δ^7^Li_serum_ values. As illustrated in Fig. 3c, d, serums are significantly depleted in the heavier ^7^Li^+^ isotopes relative to the urine, which is likely a result of reabsorption in renal tubules (Fig. S15). Balter’s mammalian model supports this conclusion [35], showing that the kidney exhibits lighter δ^7^Li values compared to serum, while other organs (brain, liver, muscle) are significantly heavier. As time following ingestion increases, the δ^7^Li_serum_ gradually decreases due to Li^+^ loss in urine via the kidneys (Fig. 2d). This explains why consistent lighter δ^7^Li_serum-12h_ values were observed in all subjects. However, it is important to note that the removal of Li^+^ by the kidneys cannot alone fully explain the difference between the SZ and BD patients, since there is no difference in the *C*_Li-normalized_.

Brain parenchyma and cerebrospinal fluid (CSF) are two another important Li^+^ reservoirs in homeostasis [43], the exchange of Li^+^ between these two reservoirs and blood stream also potentially changes the signatures of δ^7^Li_serum_. Unlike other organs and tissues, the passage of Li influx into the brain parenchyma and CSF requires traversal through the blood brain barrier (BBB) and blood-cerebrospinal fluid barrier (BCSFB), which inhibits the influx rate of Li^+^ and potentially influence Li isotopic fractionation during this process [55]. Previous research has indicated that Li can traverse the endothelial cells of the BBB through Na-coupled transporters [55], including Na^+^/H^+^ exchangers (NHE1 and NHE5). The BSCFB is known to be more permeable to certain small cations and water compared to the BBB due to the larger intercellular spaces of epithelial cells. Consequently, when Li crosses the BSCFB, it is more likely to utilize both Na-coupled transporters and paracellular diffusion pathways. Studies by Poet et al. [42] have shown that the active transmembrane transport of Li based on Na-coupled transporters is a dynamic process in which ^6^Li^+^ has a higher diffusivity compared to ^7^Li^+^. Therefore, it is speculated that the Li^+^ breakthrough the BBB/BSCFB is depleted in ^6^Li^+^, whether via passive or active transport [55], resulting in an enrichment of ^7^Li^+^ in the serum [42]. Notably, several studies have demonstrated that patients with BD and SZ have a high risk of BBB and BSF damage [8, 56]. This damage potentially enhances the rate of Li^+^ crossing the BBB and BSCFB, causing an increased depletion of ^6^Li^+^ in the serum of both BD and SZ patients. However, a significant upregulation of δ^7^Li_serum-12h_ was only observed in SZ patients compared to HC subjects. This implies the existence of other important biochemical pathways driving the decrease of δ^7^Li_serum_ values, particularly those related to intracellular processes associated with Li^+^.

Several studies on natural animal specimens have shown the enrichment of ^7^Li^+^ in specific organ tissues (Fig. S13), potentially contributes to the decrease in δ^7^Li_serum_ values [35, 57]. This phenomenon cannot be solely attributed to the transmembrane transport process unless there is aberrant activity in the efflux process, resulting in a larger intracellular depletion of ^6^Li^+^. In our iPSCs models derived from BD patients and HC subjects, we found that the intracellular δ^7^Li values were significantly higher than those in culture medium after 60 min of exposure and continued to increase by approximately 5‰ from 60 to 120 min (Fig. 4b). Importantly, the intracellular Li concentration showed minimal changes and even a slight increase during this period (Fig. 4a). Consequently, the enrichment of ^7^Li^+^ in organ tissues [35] and cells can not be attributed to the transmembrane transport processes. Instead, it suggests that certain intracellular processes lead to the accumulation of ^7^Li^+^.

**Fig. 6:**
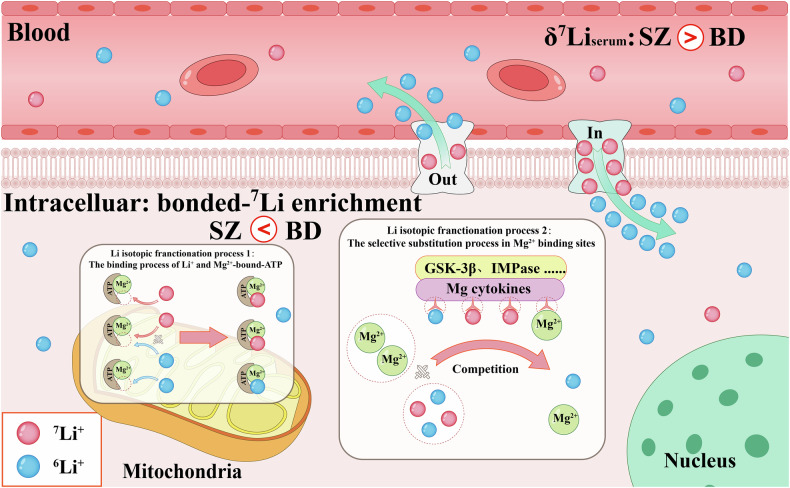
Cellular Li isotopic fractionation model. This model postulates that the intracellular competition between Li^+^ and Mg^2+^ leads to the enrichment of heavier ^7^Li isotopes, suggesting the negative shift of δ^7^Li_serum-12h_ values caused by intracellular processes. The schematic illustration of Li–Mg bridging and competition was adapted based on references [39, 40, 60].

It is well recognized that changes in the elemental bonding environment can lead to significant isotopic fractionation [58], with heavier isotopes typically enriched in stronger bonded compounds [59]. Several computational studies have demonstrated the substitutions of Mg^2+^ with Li^+^ in various Mg^2+^-bonded compounds [40, 60]. These substitutions selectively occur in specific intracellular Mg^2+^ enzymes containing highly cationic Mg^2+^-binding sites [30, 61], such as inositol monophosphatase (IMPase) and GSK-3β [40, 60]. Additionally, Li^+^ tends to form bonds with Mg^2+^-bonded ATP ([ATP-Mg-Li]^2−^) [39]. Given that mitochondria serve as the primary intracellular accumulation site of Mg^2+^ and ATP, it is likely that they play a crucial role in intracellular Li isotopic fractionation [62]. Notably, we found a strong linear relationship (R^2^ = 0.991) between the reported mitochondrial contents of different mammalian organs and Balter’s δ^7^Li data of mammalian model (Fig. S16). Furthermore, the lack of a significant difference in δ^7^Li values between red blood cells and serum is also consistent with the absence of mitochondria and nuclei in the red blood cells [35]. Moreover, the correlation observed between serum Mg concentration and *C*_Li-normalized_ (Fig. 2e) as well as δ^7^Li_serum-12h_ (Fig. 2f) also indicates that the metabolic behavior of Li concentrations and isotopic fractionations in homeostasis are closely related to Mg^2+^. The positive correlation between serum Mg concentration and *C*_Li-normalized_ may suggest that a higher rate of intracellular bonded-Li^+^ formation, competing with Mg^2+^, leads to an increase of intracellular free-Mg^2+^, as well as in the serum, and slows down the rate of Li^+^ excretion from the body (Fig. 2e). Furthermore, the negative correlation between serum Mg concentration and δ^7^Li_serum-12h_ indicates that the formation of bonded-Li^+^ during the competition between Li^+^ and Mg^2+^ may contribute to the decrease in δ^7^Li_serum_ and the significant difference of δ^7^Li_serum-12h_ between BD and SZ patients (Fig. 2f). These pieces of evidence support these substitution processes and bond formations likely lead to the enrichment of ^7^Li^+^ in cells (Fig. 6).

Extensive and diverse literature indicates that the pathophysiology of SZ is related to deficits of bioenergetic function, which differ from those observed in HC and BD [63]. These deficits are associated with the loss of motivation, a typical negative symptom stemming from impaired mitochondrial function, potentially reducing the efficacy of Li^+^ binding to [ATP-Mg]^2−^ [64]. Based on our speculation in the previous paragraph, the reduced efficacy of this binding process potentially decreases the enrichment of ^7^Li^+^ isotopes in intracellular bonded-Li⁺ in SZ, leading to further upregulation δ^7^Li values of free-Li⁺ in cellular and serum compared to HC and BD.

Furthermore, Li^+^ has been found to effectively restore the psychosocial functions for BD, while it only demonstrates a preventive effect against suicide in SZ. It has been reported that intracellular Li^+^ targets are selectively effective in BD, such as abnormally hyperactive phosphatidylinositol signaling and GSK-3β activity, which are related to Mg^2+^-bonded enzymes (IMPase and GSK-3β) [50]. Moreover, the δ^7^Li_serum-12h_ values of LiNR-BD patients appeared to be slightly heavier than those of LiR-BD patients (Fig. S17), indicating the Li isotopic fractionation is associated with the interaction of Li^+^ with intracellular Mg^2+^-bonded targets. In other words, this result also supports the correlation between the restoration (bonded-Li^+^ generation) of abnormal pathways in BD and the decrease in δ^7^Li_serum-12h_. Thus, we speculate that BD patients may exhibit heavier δ^7^Li values for intracellular bonded-Li^+^, contributing to lighter δ^7^Li_serum-12h_ for BD patients (more bonded-Li^+^) compared to those with SZ patients (Fig. 6). In summary, the compromised mitochondrial function and the reduced/absence of intracellular Li^+^ targets in SZ patients likely contributes to the significant difference of δ^7^Li_serum-12h_ between SZ and BD.

Overall, we identified significant variations in δ^7^Li_serum-12h_ among typical patients with BD and SZ after a period of Li medication, indicating its potential usefulness as a specific biomarker for psychiatrist to differentiate BD and SZ. Given that BD patients are more likely to use Li drugs, this biomarker is particularly valuable for distinguishing SZ patients who is misdiagnosed as BD patients in the initial diagnosis. Our investigation into the Li isotopic fractionation in homeostasis suggests that the differences in δ^7^Li_serum-12h_ between BD and SZ patients likely result from the intracellular selective binding processes involving competition between Li^+^ and Mg^2+^, resulting in the preferential intracellular ^7^Li^+^ enrichment. Notably, the δ^7^Li_serum-12h_ also exhibits significant differences between LiNR-BD and LiR-BD patients, although further investigation is necessary due to the limited number of LiNR-BD patients. This study holds significant implications for the field of metal medicine, and further exploration of metal isotopic fractionations could provide invaluable insights into psychiatric and other diseases, such as Alzheimer's disease [65, 66]. Further studies will delve into the Li isotopic fractionation at the organelle and molecular levels, utilizing theoretical models and advanced techniques to deepen our understanding of Li’s pharmacology and the pathophysiology of BD and SZ.

## Supplementary information

Supplemental Material

## Data Availability

Compilation of taxa is attached as an online appendix (10.17632/ptf8hj3x38.2). All code used to train models and analyze results can be found at http://scikit-learn.org/dev/index.html.
