# Supplementary material for: Natural lithium isotope variations in serum after lithium administration as a novel biomarker for differentiating schizophrenia and bipolar disorder
Source: Transl Psychiatry. 2025 Oct 7;15:386. doi: 10.1038/s41398-025-03627-6 (PMC12501255; doi:10.1038/s41398-025-03627-6)
Supplement: Supplementary file 1 — Supplemental Material [file 41398_2025_3627_MOESM1_ESM.docx]

**Supplementary Materials for**

**“Natural lithium isotope variations in serum after lithium administration as a novel biomarker for differentiating schizophrenia and bipolar disorder”**

Junhang Dong^1,2^, Baoliang Zhong^3,4^, Jun Yao^5^, Jing Chen^3,4^, Shuyang Li^1^, Pengju Xing^1^, Xiaoliang Zhou^3,4^, Gang Wang^3,4^, Lvyu Yang^5^, Ying Guo^3,4^, Chengyu Hu^6^, Zhijuan Duan^2^, Nicholas S. Belshaw^1,7^, Hongtao Zheng^2^, Xing Liu^1^ & Zhenli Zhu^1,2,8,*^.

**^1^** State Key Laboratory of Geomicrobiology and Environmental Changes, School of Earth Sciences, China University of Geosciences, Wuhan 430074, China

**^2^** Faculty of Material Science and Chemistry, China University of Geosciences, Wuhan 430074, China

**^3^** Department of Psychiatry, Wuhan Mental Health Center, Wuhan 430012, Hubei province, China

**^4^** Department of Psychiatry, Affiliated Wuhan Mental Health Center, Tongji Medical College of Huazhong University of Science & Technology, Wuhan 430012, Hubei Province, China

**^5^** State Key Laboratory of Membrane Biology, Tsinghua-Peking Center for Life Sciences, IDG/McGovern Institute for Brain Research, School of Life Sciences, Tsinghua University, Beijing 100084, China

**^6^** School of Computer Science, China University of Geosciences, Wuhan 430074, China

**^7^** Retired, Oxford University, Oxford, OX1 3PR, UK

**^8^** Hubei Key Laboratory of Yangtze Catchment Environmental Aquatic Science, Wuhan 430078, China

This Supplementary Materials contain seven Supplementary Text, Sixteen Supplementary Fig.s, three Supplementary Tables, and Supplementary References.

**1. Supporting Text**

**Supplementary Materials and Methods**

**(1) Reagents and Solutions.** Ultrapure water (UPW, >18.2 MΩ cm) utilized was obtained from a Milli-Q water purification system (Millipore gradient, Merck Millipore, Darmstadt, Germany). Prior to use, all acids, including nitric acid (HNO_3_), hydrochloric acid (HCl), and hydrofluoric acid (HF), underwent a double purification process using a Savillex's DST-1000 sub-distillation system. The acids used for the column elution were prepared by gravimetric dilution and their exact concentration were determined through titration using Na_2_CO_3_.

**(2) Cell Culture and Exposure to Natural Lithium.** The cells using in this work were derived from induced pluripotent stem cell (iPSC) lines obtained from health individuals and patients with BD, as detailed by Mertens.^1^ These iPSCs were recovered and transferred to plates coated with Matrigel (Corning, USA) and cultured in PGM1 medium (Cellapy, Cat: CA1007500, Lot: I20H197) at 37℃ and 5% CO_2_ with the medium being refreshed daily. Prior to digestion, the cells were rinsed with PBS (pH 7.4) free of CaCl_2_ and MgCl_2_ (10010-015; Gibco). The cells were passed using 0.5 mM EDTA (50 mL PBS + 35 μL EDTA) at a 1:3 split ratio every 3–4 days during culture prior to Li exposure experiments. Once the cells covered the plates, the culture medium was replaced with fresh medium containing Li (L-SVEC, δ^7^Li = 0‰) at a concentration of 20 μg mL^−1^. Blank controls were also included in the exposure experiment. After Li exposure, the culture plate was tilted at a 45° angle, and the medium was removed within 10 seconds using a low-pressure suction system (as shown Fig. S1). The volume of residual culture medium and the absolute amount of Li in this process were evaluated by analyzing the blank plate (coated with Matrigel) without additional cells (as shown Fig. S2). Subsequently, 1 mL of Accutase (Invitrogen) was added to digest the cells, and the plate was placed in an incubator (37℃ and 5% CO_2_) for 6 minutes. Then, 3 mL of DMEM/F12 medium (Gibco Life Science, Great Island, NY) was added to terminate digestion. The solution in the plate (total 4 mL) was transferred to a 15 mL centrifuge tube, and the cells were dispersed into a single cell state by backwashing the tube 5 times. The live and dead cell numbers in the plate were manually measured by Trypan Blue uptake and light microscopy. The entire cell culture and exposure to natural Li were conducted at the State Key Laboratory of Membrane Biology, Tsinghua-Peking Joint Center for Life Sciences, McGovern Institute for Brain Research, School of Life Sciences, Tsinghua University. Li-exposed cell samples were completely transferred to collection tubes, and after digestion and chemical purification, the Li concentration and δ^7^Li information of the entire cells were measured.

**(3) Sample Digestion and Chemical Purification.** Sample digestion and chemical purification were carried out in a clean laboratory at the State Key Laboratory of Geomicrobiology and Environmental Changes (GMEC), China University of Geosciences (Wuhan). Serum, urine, and cell medium samples pf different volumes (0.3/0.5/4.0 mL) were placed in a PTFE-lined stainless-steel bomb. For serum and urine, 2.0 mL concentrated HNO_3_ was slowly added, while cell medium samples required prior evaporation to dryness at 105℃ before adding HNO_3_. The sealed bombs were then heated to 190℃ in an electric oven for 12 hours. After cooling, the solutions were transferred to PFA beakers and evaporated on a hotplate at 105℃. Subsequently, 0.2 mL DI-water was added and evaporated to dryness at 105℃ to remove HNO_3_, and the remaining residue was redissolved in 8 mL 0.28 M HCl.

The Li purification was conducted using a modified one-step ion chromatographic procedure based on the work of Van Hoecke et al.^2^. The procedure involved using 8 mL of the AG 50W-X8 cation exchange resin (200–400 mesh) and a boron-silicate glass column. Prior to sample loading, the resin was cleaned and conditioned. Subsequently, 8 mL sample solution prepared with 0.28 mol L^−1^ HCl was loaded on the resin. Cations were gradually eluted at the rate of ≈ 3 min mL^−1^. After sample loading, 0.28 mol L^−1^ HCl was used to elute the sample, with the first 24 mL of eluate discarded. Then, the 25^th^ mL of eluate was collected in a 10-mL centrifuge tube and diluted using UPW to monitor Li breakthrough. The following 31 mL (26^th^–56^th^) of eluate was collected as the Li fraction, and the 57^th^ mL was also collected to check the recovery (as shown in Fig. S4). To ensure accurate Li isotope analysis, the elemental content in the eluants was monitored. The validity of the purification procedure was confirmed using two geological samples (NIST 1400 and BHVO-2) as well as two serum samples (Seronorm serum L-2 RUO and Serum SL010). The collected Li fraction solution (31 mL) was processed and diluted using 2% HNO_3_ prior to the MC-ICP-MS measurements.

**(4) Lithium Isotopic Composition Measurement Process.** The δ^7^Li values were measured using MC-ICP-MS (Nu Plasma II, Nu instruments, UK). The analytical sensitivity was checked prior to analysis, and all measurements were carried out under daily optimized settings. A 100 ng mL^−1^ Li solution consistently produced signal intensities exceeding 1.5 V on ^7^Li, thereby ensuring reliable and high-precision isotopic ratio determinations. The purified Li solutions (200 ng mL^−1^ Li in 2% HNO_3_ (v/v)) were introduced using a MicroMist low-flow nebulizer (100 μL min^−1^, Glass Expansion) and a quartz glass spray chamber coupled with an auto-sampler. Faraday cups of H9 and L6 were utilized to measure ^7^Li and ^6^Li simultaneously. The results were reported as δ^7^Li = [(^7^Li/^6^Li _sample_) / (^7^Li/^6^Li _standard_) − 1] × 1000‰ relative to the Li isotope standard (L-SVEC). To determine the accuracy of the established analytical procedure for purification and measurement of Li isotopes, we firstly measured three international geological CRMs. As shown in Fig. S5a, the determined δ^7^Li values of BHVO-2 (4.2 ± 0.4‰), BCR-2 (2.9 ± 0.4‰), and SRM-1400 (−1.8 ± 0.4‰) CRMs were in excellent agreement with the published values of 4.3 ± 0.3‰,^3^ 2.7 ± 0.4‰,^4^ and −1.7 ± 0.5‰.^5^ To further validate the accuracy of the procedure for serum samples, we carried out multiple-replicate measurements of three serum CRMs (Fig. S5b). The determined δ^7^Li values were 18.7 ± 0.5‰ (n = 11, 2SD), 18.5 ± 0.6‰ (n = 21, 2SD), and −7.2 ± 0.4‰ (n = 13, 2SD) for Seronorm Serum L-1, Seronorm Serum L-2, and BCR-304, respectively. Notably, the δ^7^Li values of the three serum CRMs exhibited a considerable fractionation difference of up to 25‰. During the analytical session, routine analysis included the measurement of at least one reference materials (Seronorm serum L-1/L-2 RUO) and one inter-lab standard Li solution. Long-term external reproducibility was found to be better than ± 0.3‰ (2SD, n = 150) for in-house standard solutions.

**Lithium Isotopic Compositions in Li Drugs with Different Brands.**

For patients prescribed Li medication, over 99% of the Li presented in their bodies originates from the prescribed Li drugs. Therefore, the interference from diet is negligible, but it is crucial to evaluate the differences between brands and batches of Li drugs. Our measurements revealed significant differences of δ^7^Li values (from +3.2‰ to +8.3‰) in Li drug (δ^7^Li_drug_) among different brands (Figure. S3). This information underscores the importance of ensuring a consistent brand of Li medication for this study. All patients and healthy volunteers received Enhua Li medication, the exclusive brand of Li drugs used in this research.

**The Variation of Normalized Serum Li Level.**

No significant correlations were found between serum Li concentrations and various anthropological parameters, including sex, age, height, weight, BMI, and daily Li_2_CO_3_ dosage (Fig. S9 a, c and Fig. S10 a–l). However, a marginal relationship was observed between Li_2_CO_3_ dosage per unit body weight and serum Li concentrations in both BD (Figure S10 f) and SZ patients (Figure S10 l). Therefore, the normalized serum Li levels (*C*_Li-normalized_) was defined as the serum Li concentration (mg kg^−1^) divided by the Li dosage per unit body weight (mg kg^−1^), which showed no significant differences between BD and SZ (0.0265 ± 0.0131 *vs.* 0.0290 ± 0.0158, *P* > 0.05, Fig. S11).

**The correlation analysis between δ^7^Li_serum-12h_ and anthropometrical parameters.**

There was no significant correlation between the δ^7^Li_serum-12h_ values and the anthropometrical parameters (i.e., sex, age, height, weight, BMI, daily Li_2_CO_3_ dosage, and Li_2_CO_3_ dosage per unit body weight) in either patient group (Fig. S9 c, d and Fig. S12 a–l).

**2. Supplementary Fig.s**


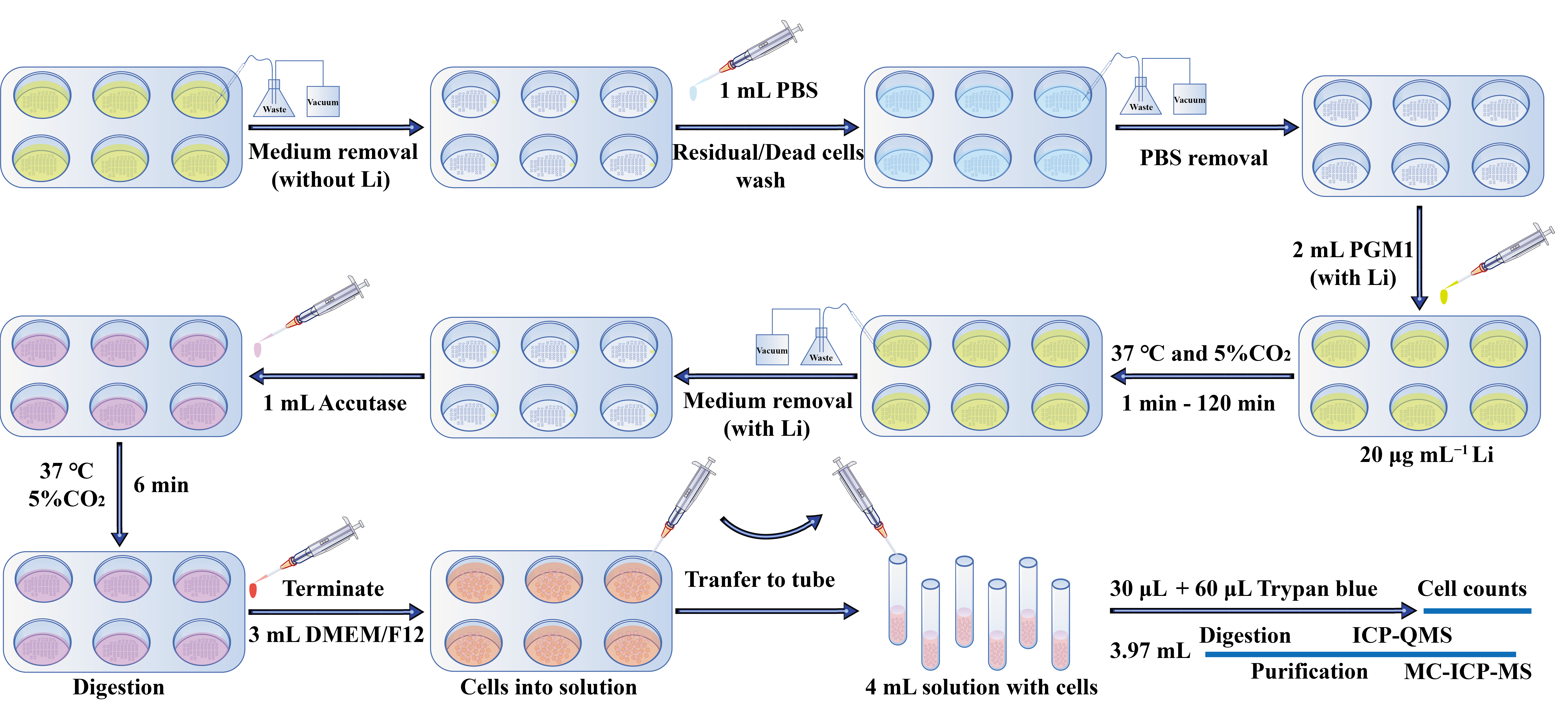


**Fig. S1** The illustration of the cells culture and collection processes.


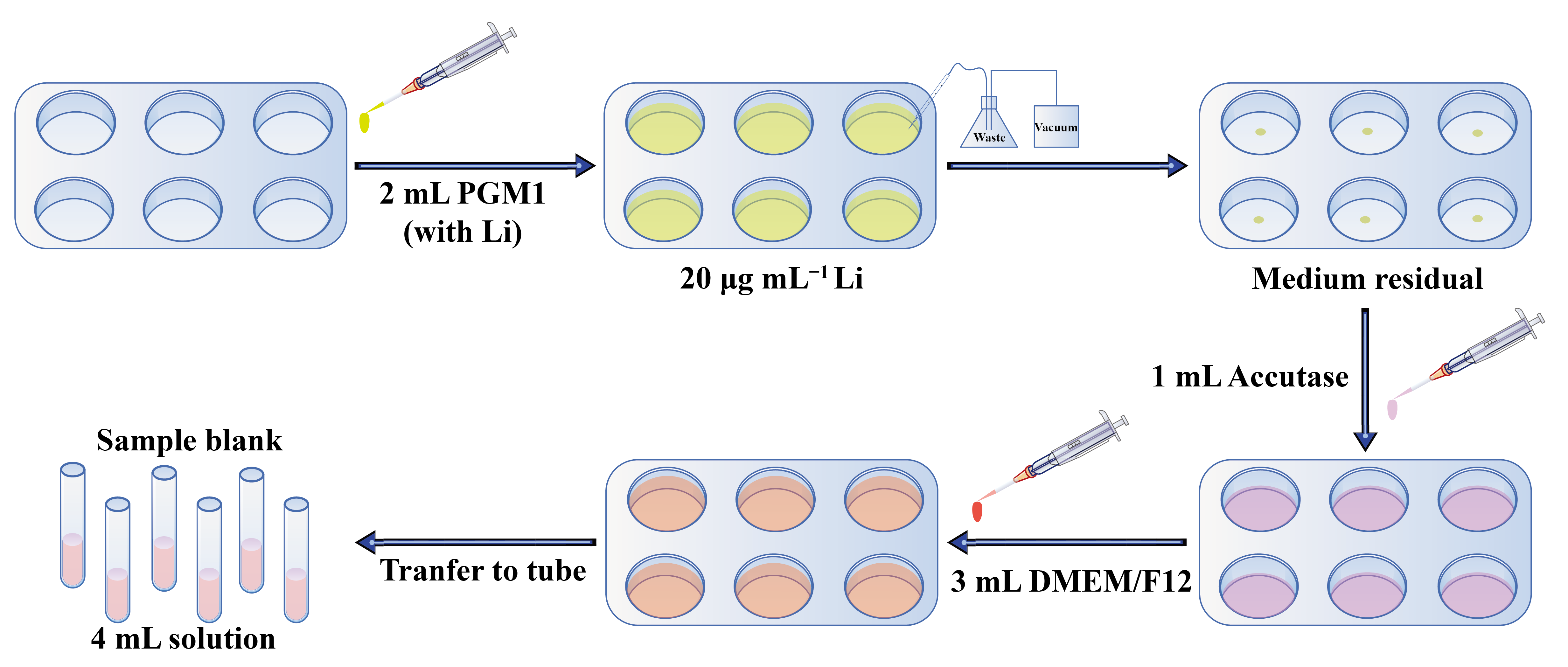


**Fig. S2** The illustration of the collection process for the samples blank.

**
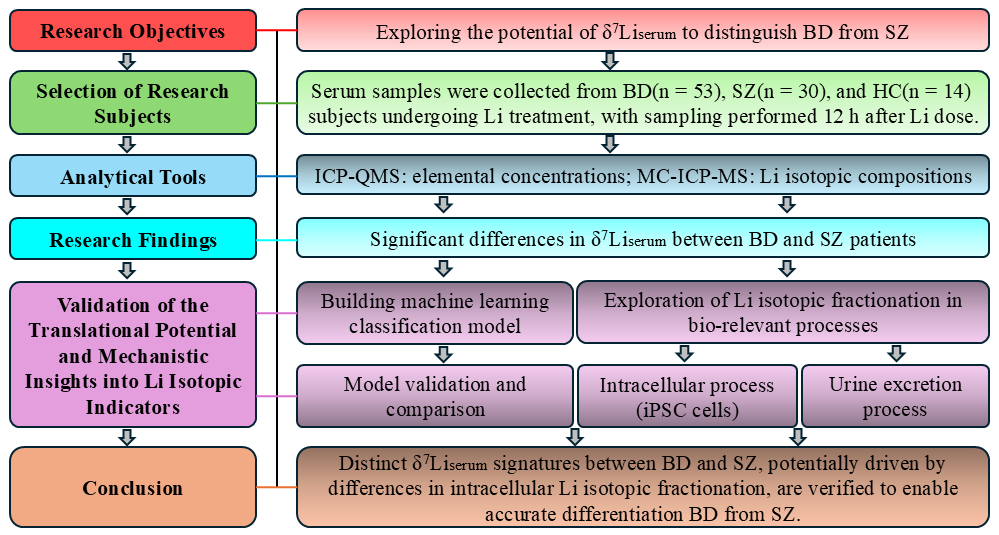
**

**Fig. S3** Schematic representation of the experimental design in this study.


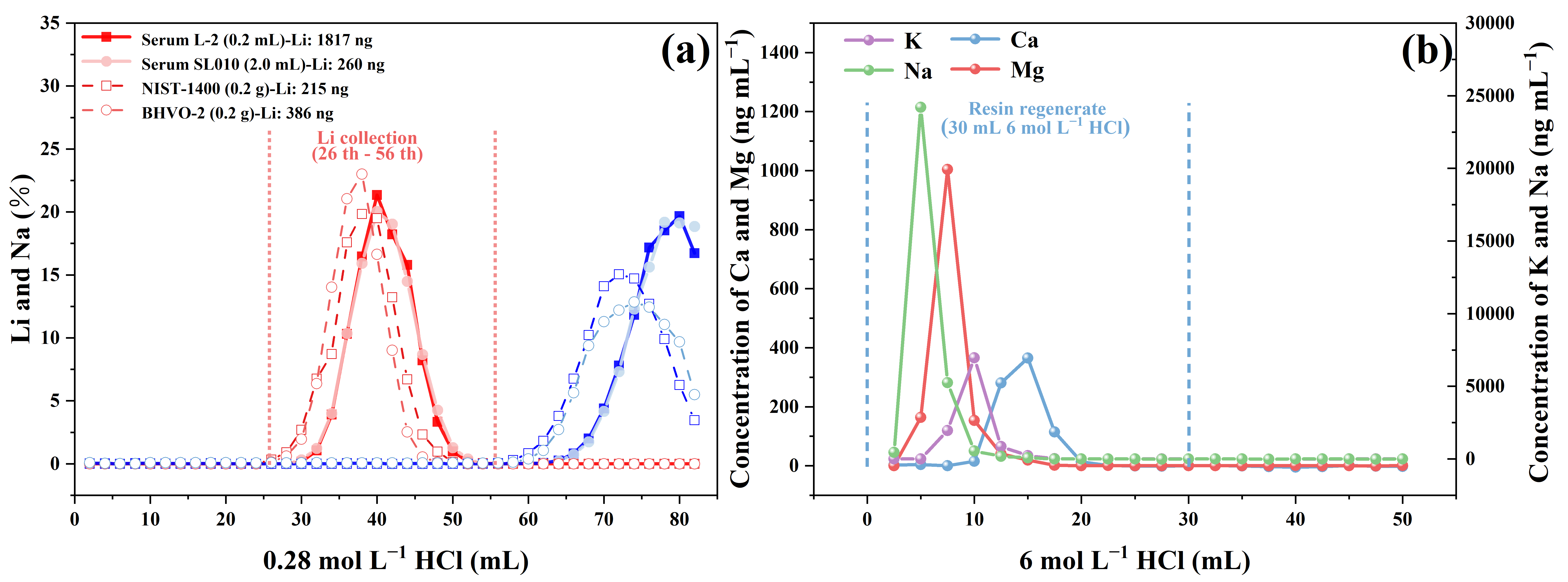


**Fig. S4** (a) Elution profiles of Li and Na for Seronorm serum L-2 RUO, serum SL010, NIST-1400, and BHVO-2. The value shown after the sample name represents the Li mass loaded on resin, and the blue section indicates Na elution; (b) Volume of 6 mol L^−1^ HCl needed for the resin regeneration after Li purification of serum SL010.


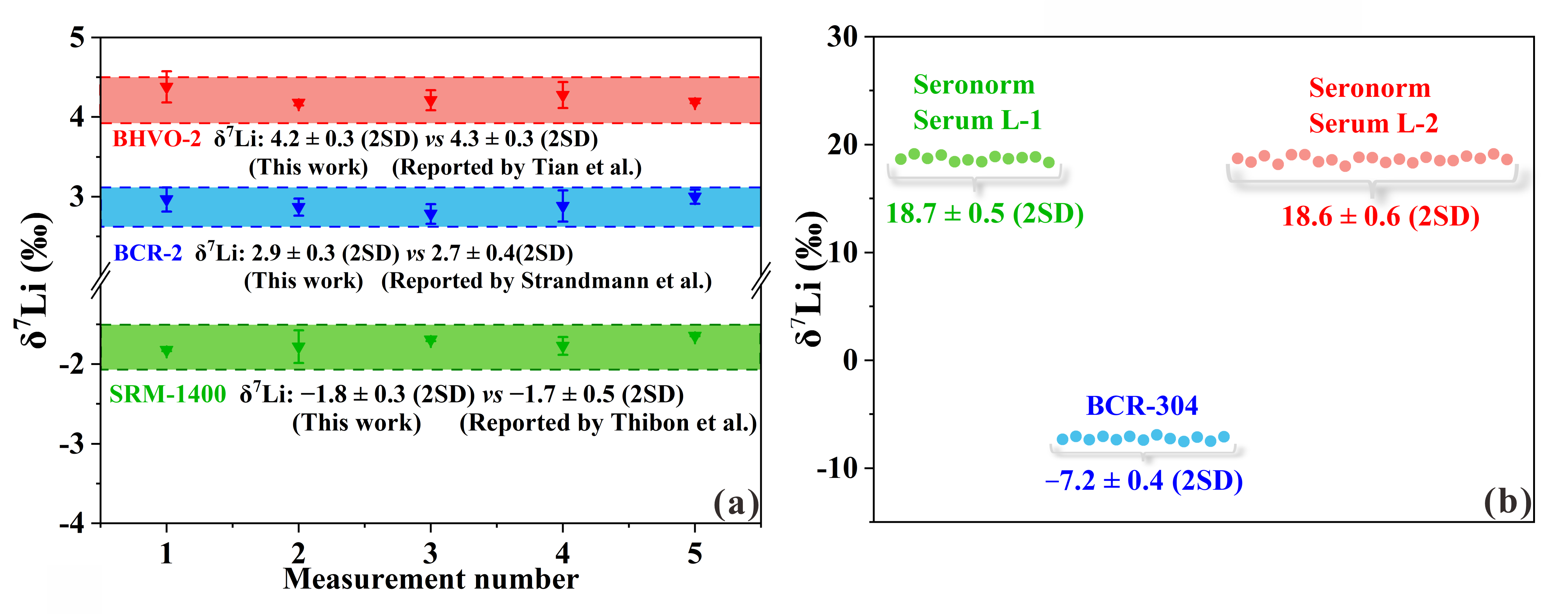


**Fig. S5** (a) The comparison of the determined δ^7^Li values of BHVO-2, BCR-2, and SRM 1400 (N = 5) with reported reference values. (b) The determined δ^7^Li values of three serum CRMs (Seronorm Serum L-1, Seronorm Serum L-2, and BCR-304). Each triangle and circle correspond to a separate Li purification. Average and 2SD (in ‰) of the determined δ^7^Li are reported below symbols.


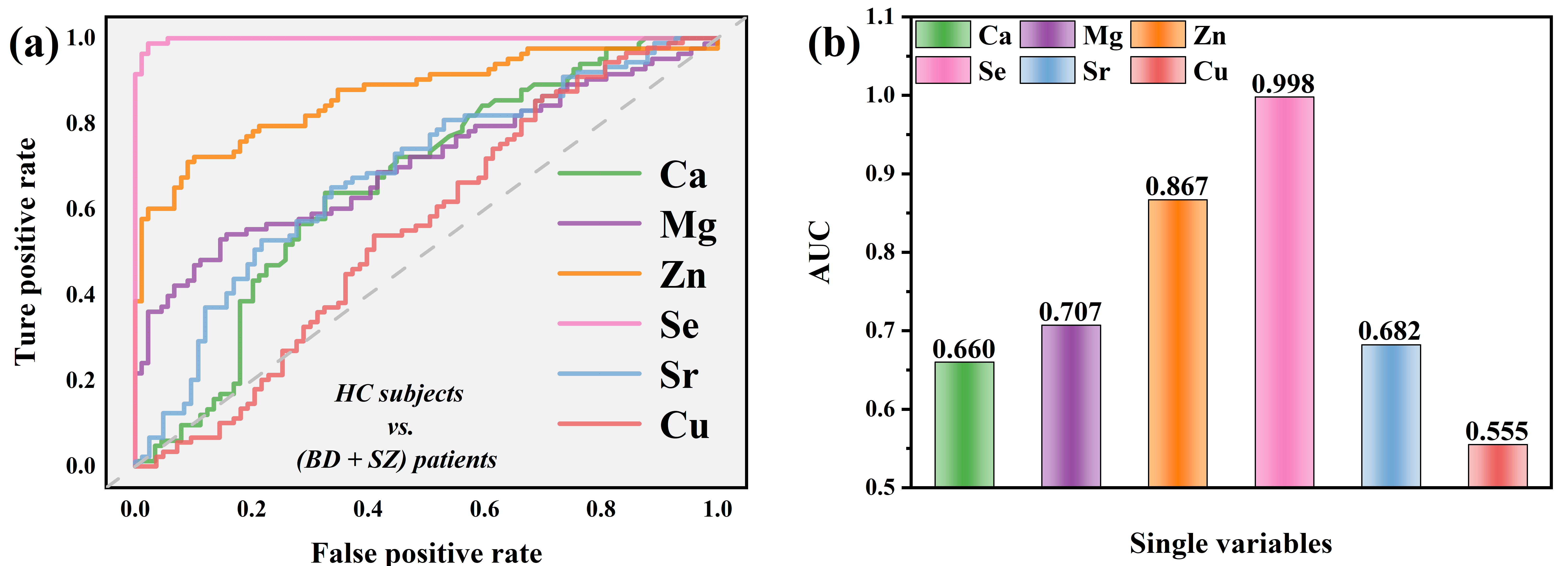


**Fig. S6** The (a) receiver-operating characteristic (ROC) curves and (b) area under the ROC curves (AUCs) of six single variables of serum Ca, Mg, Zn, Se, Sr, and Cu under the view of BD/SZ patients *vs.* HC subjects. The ROC curves were generated using true positive rate as the y-axis and false positive rate as the x-axis.


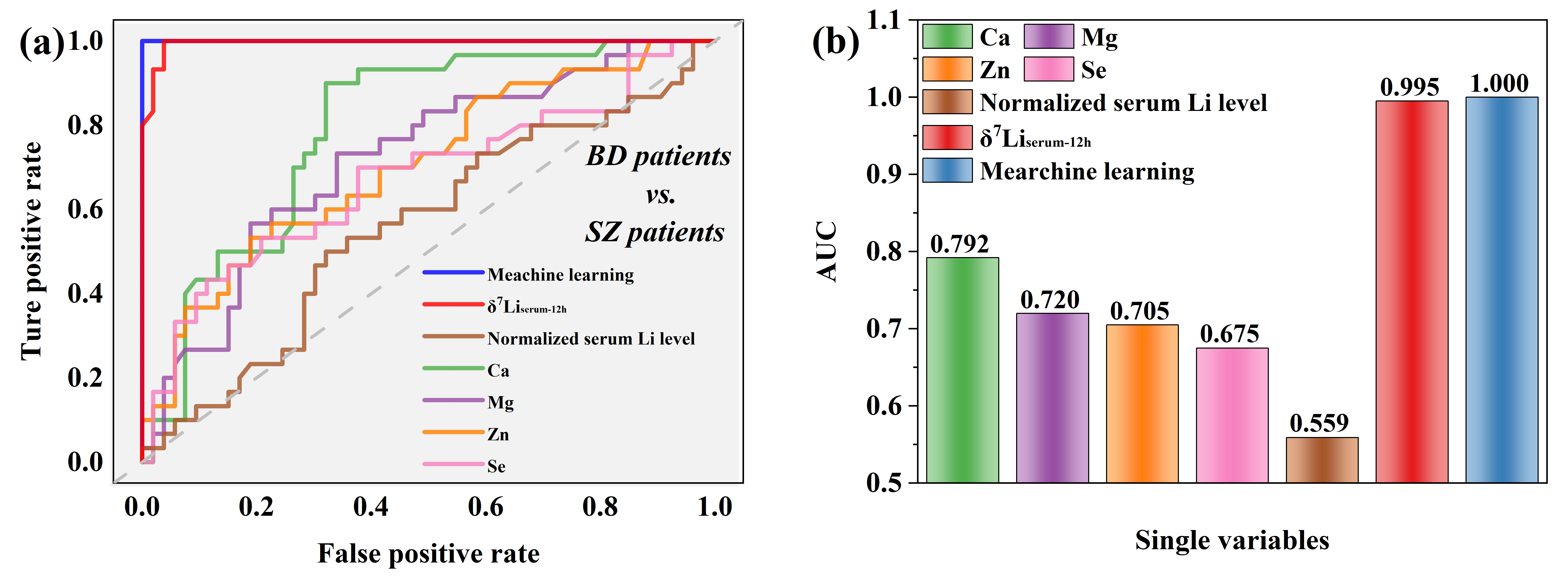


**Fig. S7** The (a) ROC curves and (b) AUCs of the machine learning model and the single variables without RF classification under the view of BD *vs.* SZ. The ROC curves were generated using true positive rate as the y-axis and false positive rate as the x-axis.


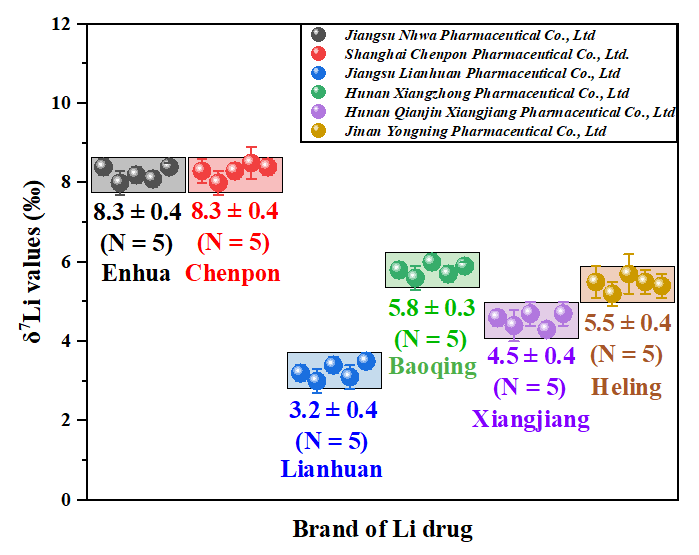


**Fig. S8** The δ^7^Li values of Li drugs with different manufacturers in Chinese domestic market, δ^7^Li measurements were conducted with five tablets for each brand.


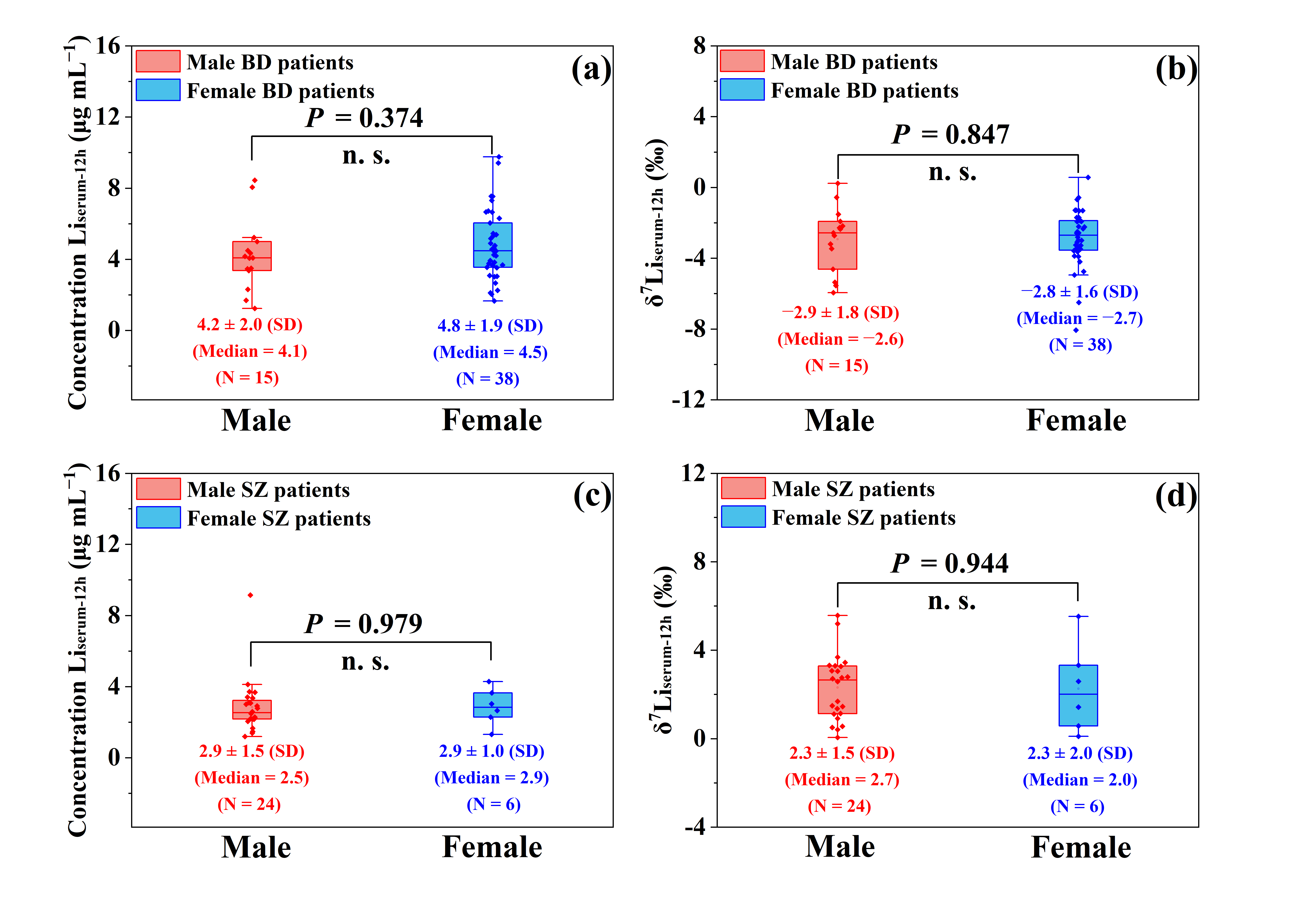


**Fig. S9** The comparison of serum Li concentrations and δ^7^Li_serum-12h_ values between male and female patients with BD (a, b) and SZ (c, d).


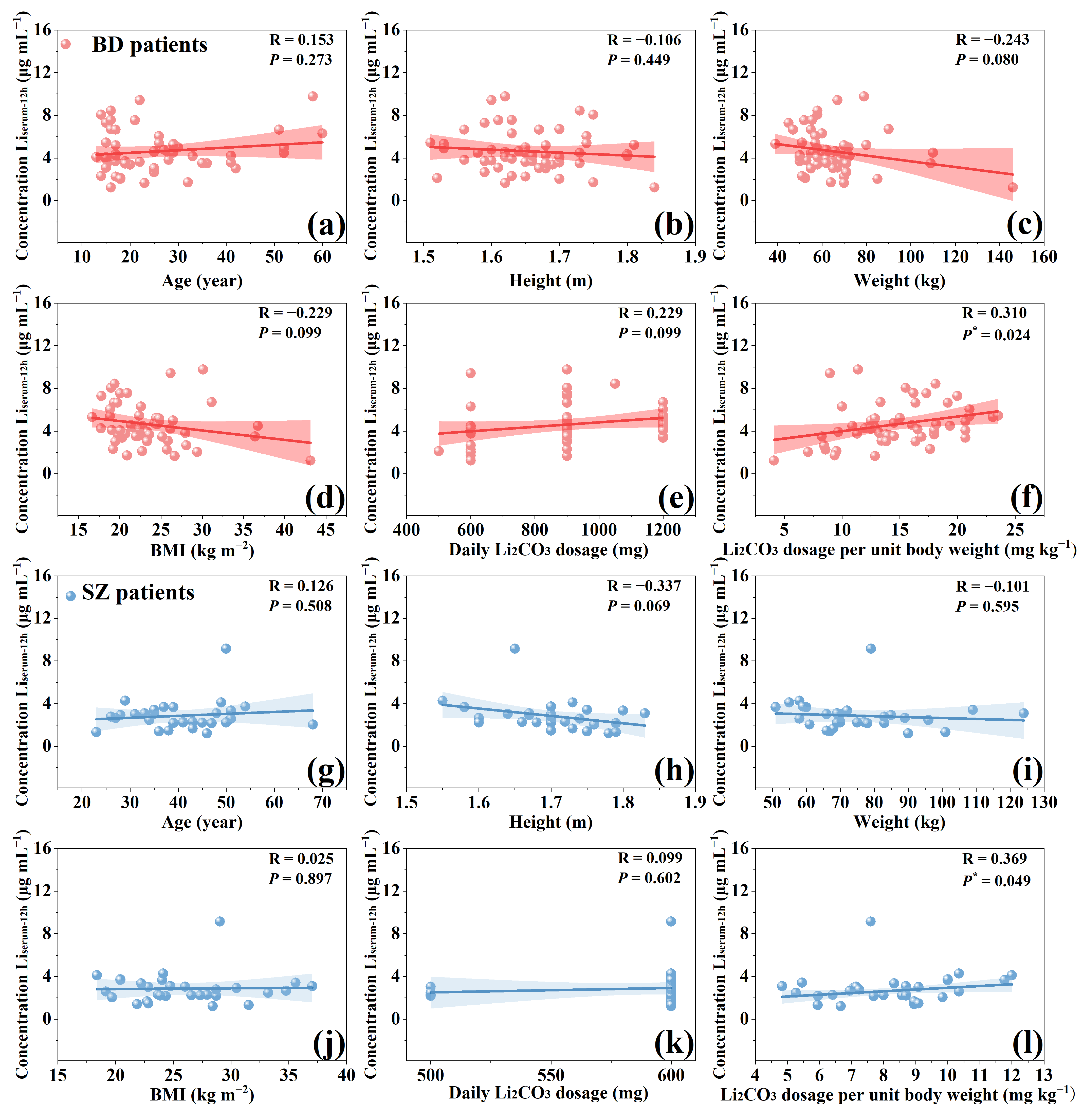


**Fig. S10** Statistical results showing the correlation between anthropological parameters (age, height, weight, BMI, daily Li_2_CO_3_ dosage and Li_2_CO_3_ dosage per unit body weight) and Li concentrations in serum from BD patents (red, a–f) and SZ patients (blue, g–l).


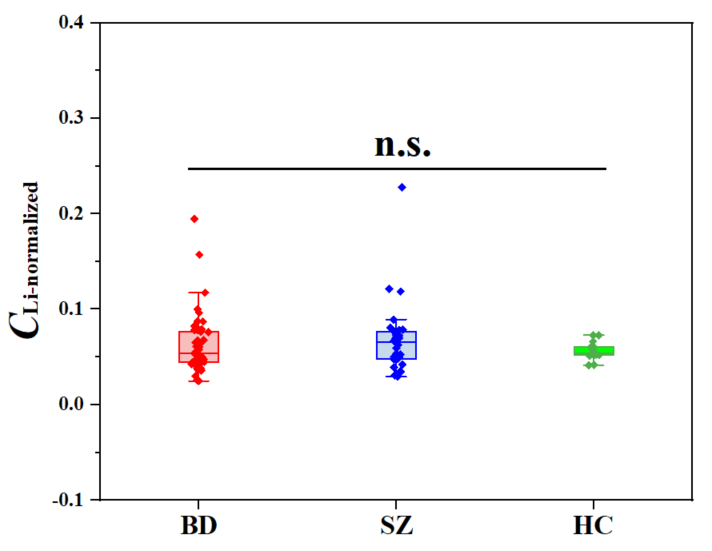


**Fig. S11** The comparison of the *C*_Li-normalized_ after 12 hours of Li drug intake between HC subjects (green box, N = 14), BD patients (red box, N = 53) and SZ patients (blue box, N = 30). The data are presented as box-and-whiskers graphs, with the box extending from the 25th to 75th percentiles, and the horizontal line representing the median.


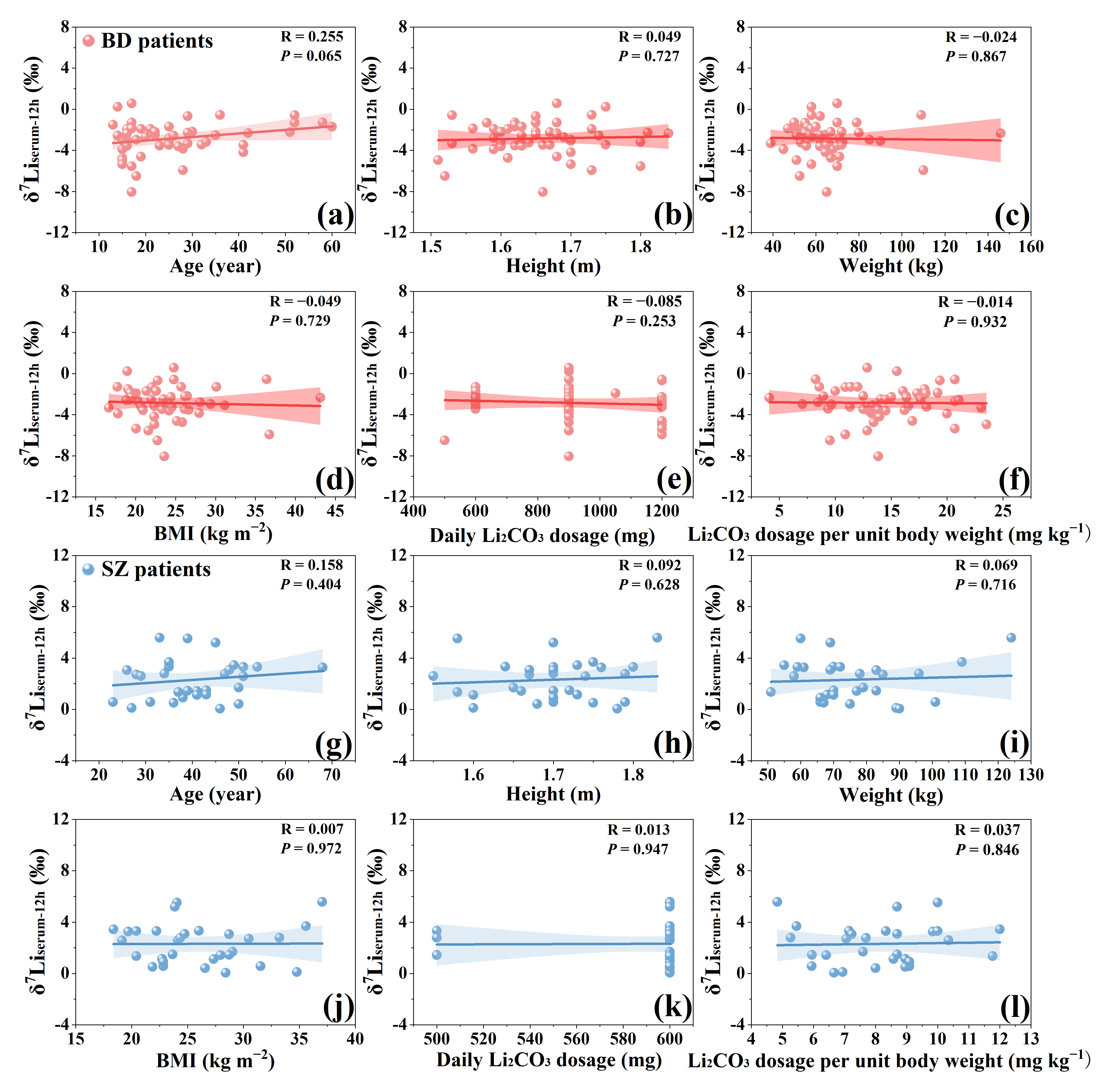


**Fig. S12** Statistical results showing the correlation between anthropological parameters (age, height, weight, BMI, daily Li_2_CO_3_ dosage and Li_2_CO_3_ dosage per unit body weight) and δ^7^Li_serum-12h_ from BD patents (red, a–f) and SZ patients (blue, g–l).

**
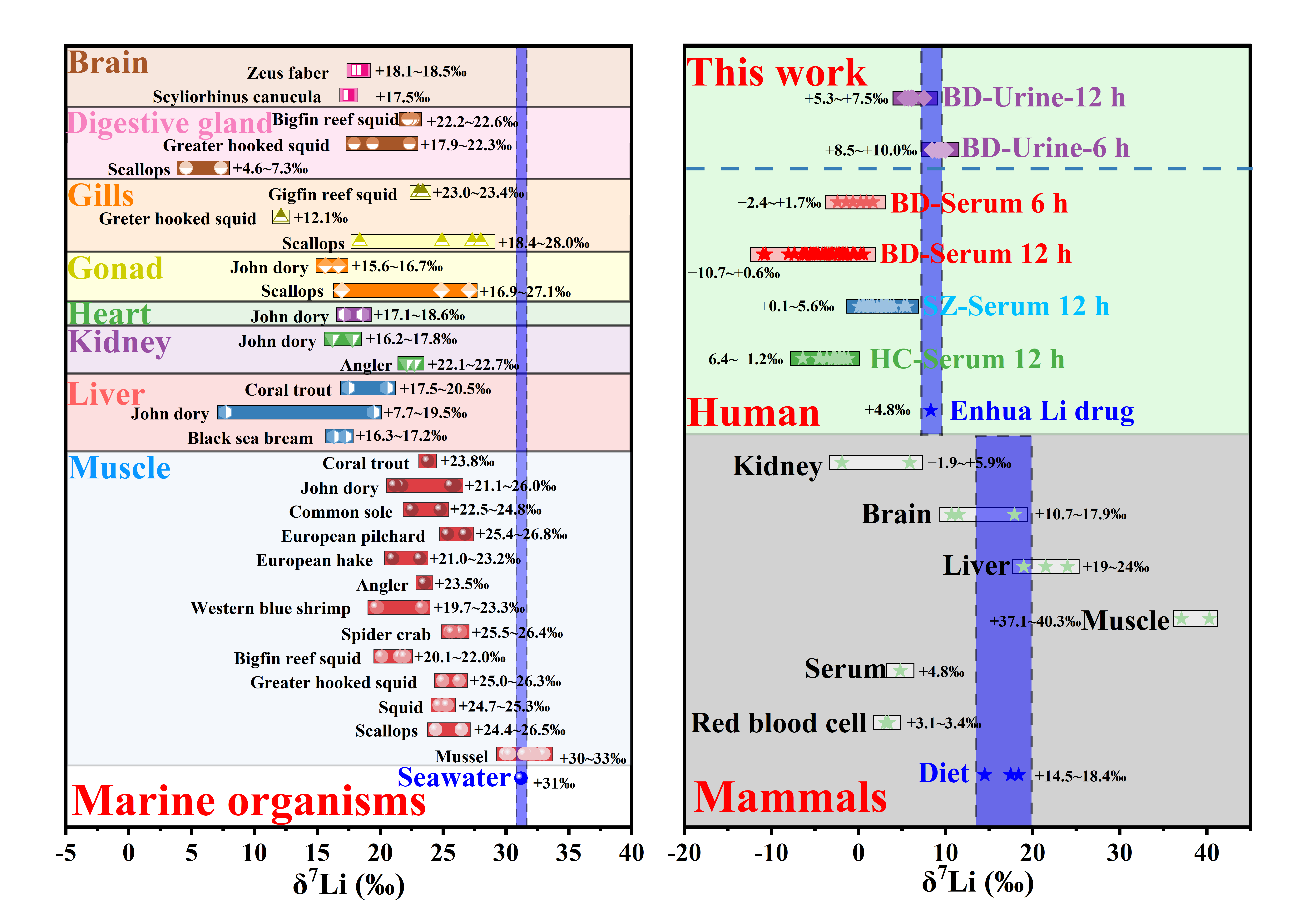
**

**Fig. S13** Reservoir map of the δ^7^Li values of modern biological tissues and body fluid samples. The δ^7^Li values of marine organisms and mammals are from previous published work.^6-8^ Each point represents one sample originating from an independent individual.

**
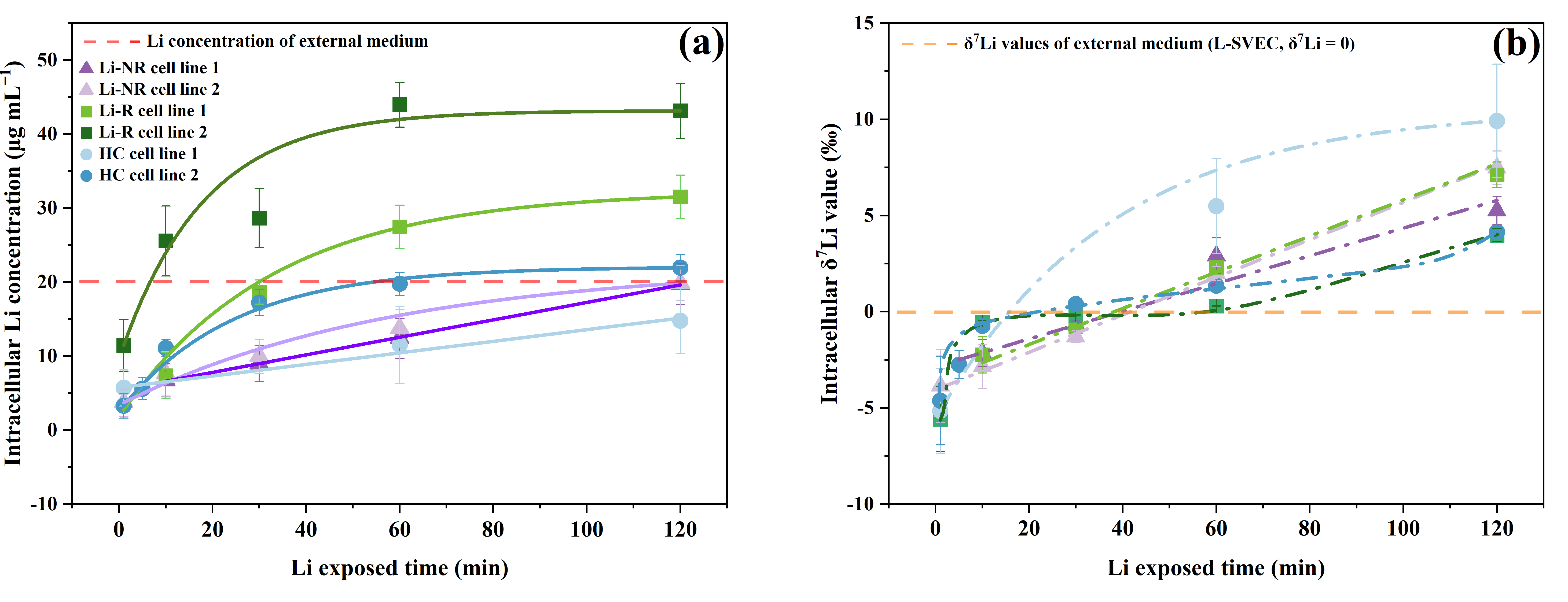
**

**Fig. S14** The intracellular (a) Li concentrations and (b) δ^7^Li values from LiNR-BD (triangle symbols and purple lines), LiR-BD (square symbols and green lines) and HC iPSCs (circle symbols and blue lines) exposed at PGM1 solution with 20 μg mL^−1^ Li (L-SVEC) from 1 min to 60 min.


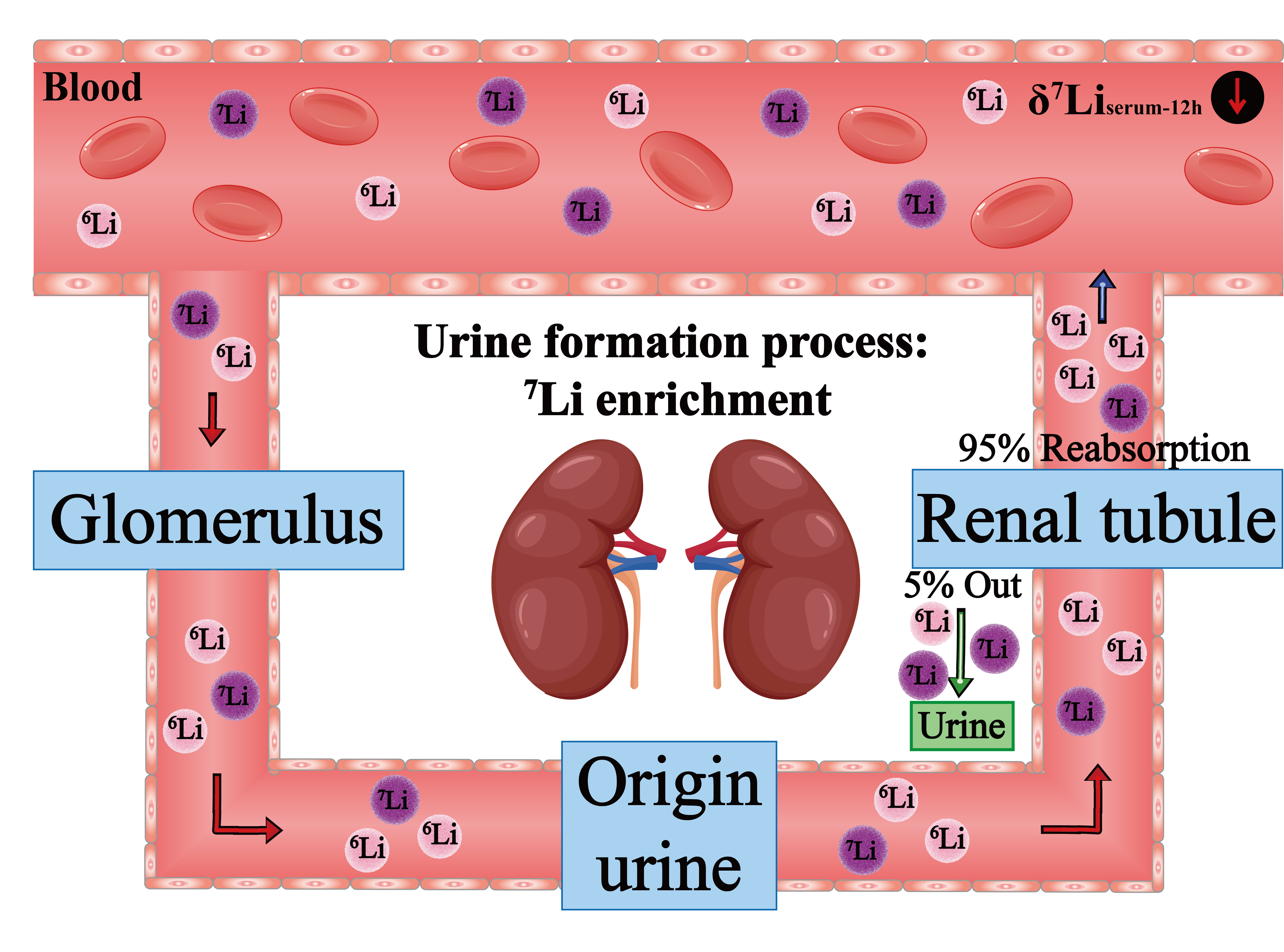


**Fig. S15** Schematic diagram of the Li isotopic fractionation model during urine formation and elimination.


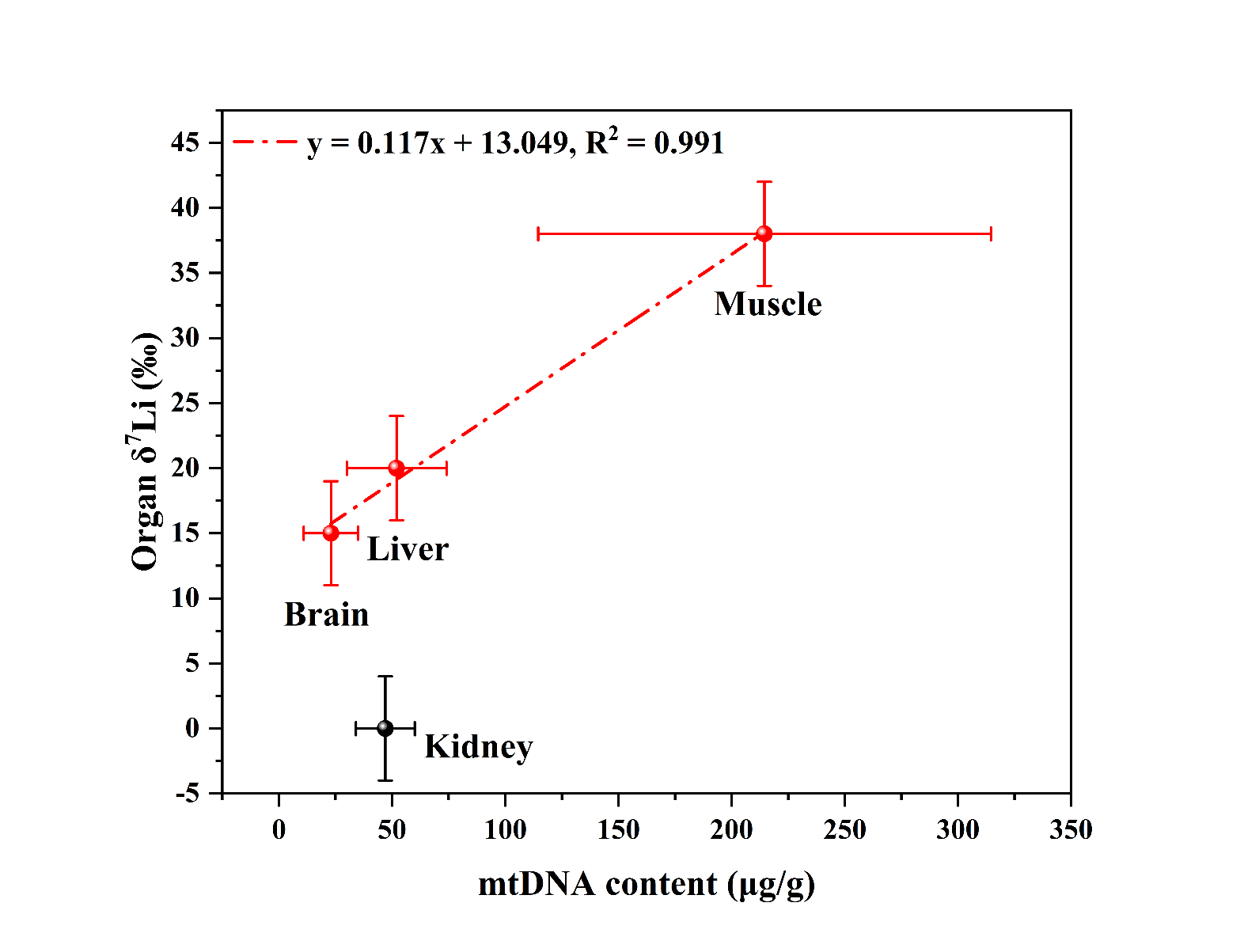


**Fig. S16** Correlation between δ^7^Li values in different types of organ tissues (data from mammalian model) and mitochondrial DNA (mtDNA) content in various types of organ tissues. The δ^7^Li values are obtained from sheep mammalian model^6^ and the mtDNA contents are sourced from mouse mammalian organs and muscles of human volunteers.^9, 10^


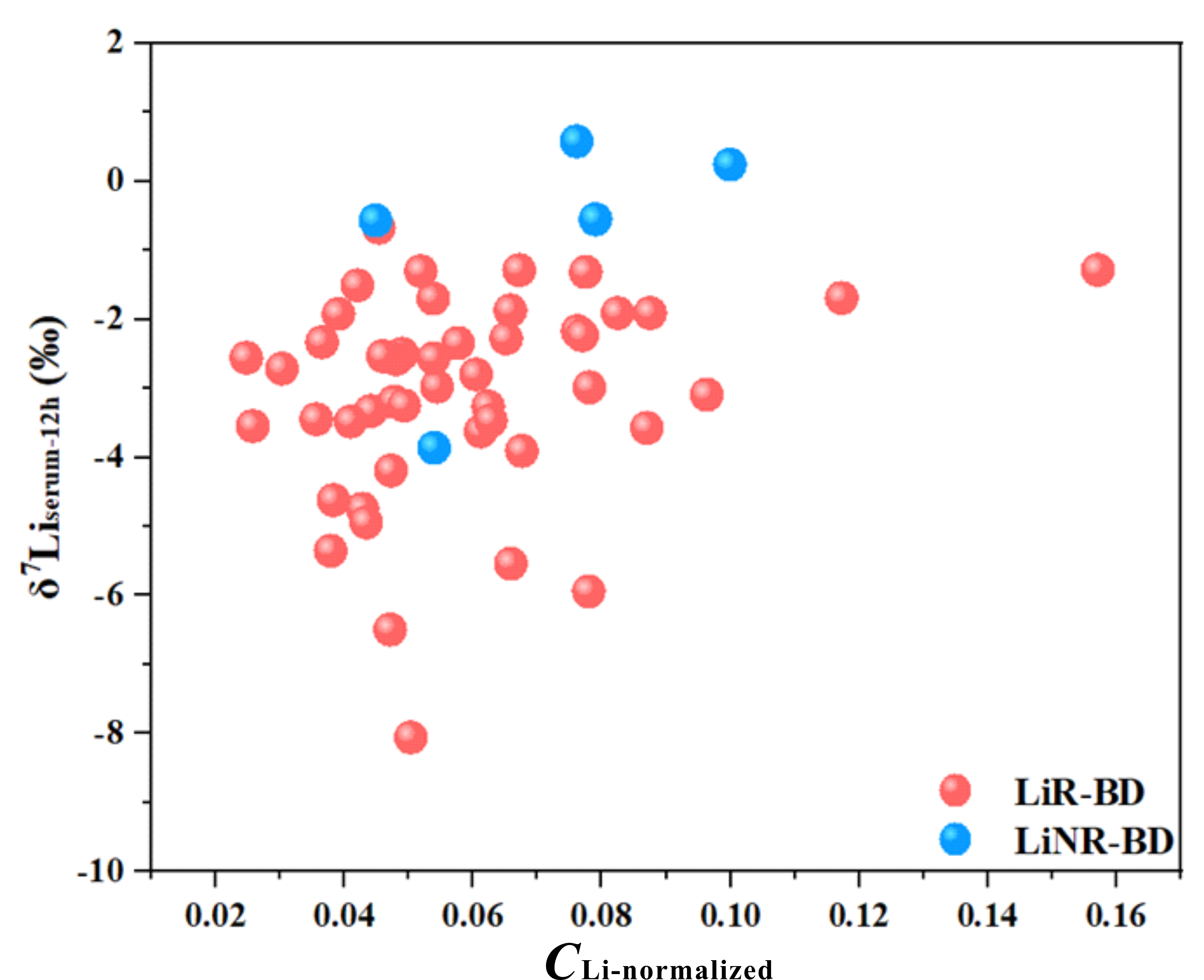


**Fig. S17** The comparison of two-dimensional information (δ^7^Li_serum-12h_ and *C*_Li-normalized_) of LiR-BD (red box, N = 48) patients and LiNR-BD patients (blue box, N = 5).

**3. Supplementary Tables**

Table S1. Detailed clinical information of BD patients.

| **Code** | **Sex** | **Age** | **Course (year)** | **Height/cm** | **Weight/kg** | **Daily dosage of Li_2_CO_3_/mg** | **Serum Li concentration (μg mL^−1^)** | **δ^7^Li_serum-12h_/‰** |
| --- | --- | --- | --- | --- | --- | --- | --- | --- |
| BD Patient 1 | Male | 28 | 10 | 173 | 110 | 1200 | 4.5 | −5.9 ± 0.1 |
| BD Patient 2 | Male | 17 | 2 | 180 | 72 | 900 | 4.4 | −5.5 ± 0.1 |
| BD Patient 3 | Male | 20 | 0.25 | 169 | 57 | 1200 | 3.4 | −2.7 ± 0.1 |
| BD Patient 4 | Female | 15 | 2 | 159 | 44 | 900 | 7.3 | −3.9 ± 0.3 |
| BD Patient 5 | Female | 29 | 1 | 153 | 40 | 900 | 5.3 | −3.3 ± 0.1 |
| BD Patient 6 | Female | 52 | 12 | 158 | 55 | 600 | 4.5 | −1.3 ± 0.2 |
| BD Patient 7 | Female | 16 | 4 | 160 | 53 | 600 | 3.8 | −3.3 ± 0.2 |
| BD Patient 8 | Female | 25 | 2 | 162 | 56 | 900 | 4.6 | −1.7 ± 0.1 |
| BD Patient 9 | Female | 16 | 5 | 170 | 91 | 1200 | 6.7 | −3.1 ± 0.2 |
| BD Patient 10 | Female | 18 | 0.5 | 152 | 59 | 500 | 2.1 | −6.5 ± 0.2 |
| BD Patient 11 | Female | 21 | 6 | 161 | 52 | 900 | 7.5 | −1.9 ± 0.3 |
| BD Patient 12 | Female | 15 | 5 | 161 | 66 | 900 | 3.1 | −4.7 ± 0.2 |
| BD Patient 13 | Male | 30 | 11 | 165 | 73 | 900 | 5.0 | −2.2 ± 0.2 |
| BD Patient 14 | Female | 22 | 5 | 165 | 64 | 900 | 3.6 | −2.5 ± 0.1 |
| BD Patient 15 | Female | 28 | 10 | 156 | 67 | 900 | 3.8 | −3.9 ± 0.3 |
| BD Patient 16 | Female | 19 | 5 | 159 | 51 | 900 | 3.7 | −1.9 ± 0.2 |
| BD Patient 17 | Female | 58 | 9 | 162 | 77 | 900 | 9.8 | −1.3 ± 0.1 |
| BD Patient 18 | Female | 17 | 8 | 165 | 73 | 600 | 2.3 | −1.3 ± 0.3 |
| BD Patient 19 | Male | 32 | 12 | 175 | 67 | 600 | 1.7 | −3.4 ± 0.1 |
| BD Patient 20 | Female | 29 | 5 | 165 | 64 | 1200 | 4.5 | −0.7 ± 0.2 |
| BD Patient 21 | Female | 23 | 5 | 162 | 74 | 900 | 1.7 | −3.5 ± 0.2 |
| BD Patient 22 | Male | 13 | 1 | 162 | 49 | 900 | 4.1 | −1.5 ± 0.1 |
| BD Patient 23 | Male | 36 | 18 | 173 | 108 | 900 | 3.5 | −0.6 ± 0.3 |
| BD Patient 24 | Female | 52 | 26 | 153 | 58 | 1200 | 4.9 | −0.6 ± 0.1 |
| BD Patient 25 | Female | 15 | 2 | 163 | 63 | 600 | 3.9 | −3.0 ± 0.1 |
| BD Patient 26 | Female | 15 | 3 | 151 | 51 | 1200 | 5.5 | −4.9 ± 0.1 |
| BD Patient 27 | Male | 19 | 6 | 168 | 70 | 1200 | 3.5 | −4.6 ± 0.2 |
| BD Patient 28 | Female | 27 | 10 | 160 | 61 | 900 | 4.8 | −3.6 ± 0.1 |
| BD Patient 29 | Female | 16 | 0.25 | 163 | 55 | 900 | 7.6 | −3.6 ± 0.2 |
| BD Patient 30 | Male | 14 | 0.5 | 175 | 59 | 900 | 8.1 | 0.2 ± 0.2 |
| BD Patient 31 | Male | 16 | 0.25 | 173 | 58 | 1050 | 8.4 | −1.9 ± 0.3 |
| BD Patient 32 | Male | 14 | 1 | 163 | 51 | 900 | 2.3 | −2.6 ± 0.1 |
| BD Patient 33 | Female | 17 | 0.5 | 166 | 65 | 900 | 3.7 | −8.1 ± 0.3 |
| BD Patient 34 | Female | 18 | 0.8 | 170 | 85 | 600 | 2.0 | −3.0 ± 0.1 |
| BD Patient 35 | Female | 17 | 4 | 168 | 50 | 600 | 4.3 | −1.3 ± 0.1 |
| BD Patient 36 | Female | 22 | 6 | 160 | 66 | 600 | 9.4 | −2.2 ± 0.1 |
| BD Patient 37 | Male | 33 | 6 | 180 | 73 | 1200 | 4.2 | −3.2 ± 0.1 |
| BD Patient 38 | Female | 17 | 5 | 156 | 47 | 900 | 6.7 | −1.9 ± 0.3 |
| BD Patient 39 | Male | 29 | 0.45 | 181 | 79 | 1200 | 5.2 | −2.3 ± 0.1 |
| BD Patient 40 | Female | 25 | 10 | 159 | 72 | 600 | 2.7 | −2.8 ± 0.1 |
| BD Patient 41 | Female | 51 | 0.75 | 167 | 55 | 900 | 6.7 | −2.2 ± 0.2 |
| BD Patient 42 | Female | 42 | 13 | 168 | 58 | 900 | 3.0 | −2.3 ± 0.1 |
| BD Patient 43 | Female | 60 | 1 | 163 | 59 | 600 | 6.3 | −1.7 ± 0.2 |
| BD Patient 44 | Female | 41 | 14 | 170 | 63 | 900 | 3.6 | −4.2 ± 0.1 |
| BD Patient 45 | Female | 41 | 23 | 166 | 71 | 900 | 4.2 | −3.5 ± 0.2 |
| BD Patient 46 | Female | 25 | 7 | 164 | 68 | 1200 | 4.6 | −3.2 ± 0.1 |
| BD Patient 47 | Female | 26 | 19 | 174 | 57 | 1200 | 6.1 | −2.6 ± 0.1 |
| BD Patient 48 | Female | 25 | 3.5 | 167 | 64 | 900 | 3.0 | −3.5 ± 0.1 |
| BD Patient 49 | Male | 16 | 1.5 | 184 | 147 | 600 | 1.2 | −2.3 ± 0.3 |
| BD Patient 50 | Male | 15 | 1 | 170 | 59 | 1200 | 4.1 | −5.4 ± 0.1 |
| BD Patient 51 | Female | 26 | 19 | 174 | 57 | 1200 | 5.4 | −2.6 ± 0.2 |
| BD Patient 52 | Female | 17 | 6 | 168 | 70 | 900 | 5.2 | 0.6 ± 0.1 |
| BD Patient 53 | Female | 35 | 3 | 168 | 62 | 900 | 3.5 | −2.5 ± 0.3 |

Table S2. Detailed clinical information of SZ patients.

| **Code** | **Sex** | **Age** | **Course (year)** | **Height/cm** | **Weight/kg** | **Daily dosage of Li_2_CO_3_/mg** | **Serum Li concentration (μg mL^−1^)** | **δ^7^Li_serum-12h_/‰** |
| --- | --- | --- | --- | --- | --- | --- | --- | --- |
| SZ Patient 1 | Male | 49 | 17 | 173 | 53 | 600 | 4.1 | 3.4 ± 0.1 |
| SZ Patient 2 | Male | 50 | 12 | 165 | 79 | 600 | 9.2 | 1.7 ± 0.1 |
| SZ Patient 3 | Male | 33 | 18 | 183 | 124 | 600 | 3.1 | 5.6 ± 0.1 |
| SZ Patient 4 | Male | 28 | 4 | 167 | 85 | 600 | 2.9 | 2.7 ± 0.1 |
| SZ Patient 5 | Female | 39 | 6 | 158 | 60 | 600 | 3.7 | 5.5 ± 0.2 |
| SZ Patient 6 | Male | 31 | 16 | 170 | 66 | 600 | 3.0 | 0.6 ± 0.1 |
| SZ Patient 7 | Female | 29 | 11 | 155 | 58 | 600 | 4.3 | 2.6 ± 0.2 |
| SZ Patient 8 | Female | 35 | 15 | 164 | 70 | 500 | 3.0 | 3.3 ± 0.1 |
| SZ Patient 9 | Male | 39 | 6 | 170 | 84 | 500 | 2.2 | 1.5 ± 0.1 |
| SZ Patient 10 | Female | 41 | 8 | 166 | 78 | 500 | 2.3 | 1.4 ± 0.2 |
| SZ Patient 11 | Male | 34 | 9 | 170 | 96 | 500 | 2.5 | 2.8 ± 0.2 |
| SZ Patient 12 | Male | 35 | 10 | 175 | 110 | 600 | 3.4 | 3.7 ± 0.2 |
| SZ Patient 13 | Female | 27 | 8 | 160 | 88 | 600 | 2.7 | 0.1 ± 0.1 |
| SZ Patient 14 | Male | 51 | 15 | 174 | 58 | 600 | 2.6 | 2.6 ± 0.5 |
| SZ Patient 15 | Male | 50 | 30 | 168 | 75 | 600 | 2.2 | 0.4 ± 0.4 |
| SZ Patient 16 | Male | 68 | 46 | 176 | 61 | 600 | 2.0 | 3.3 ± 0.2 |
| SZ Patient 17 | Male | 46 | 27 | 178 | 90 | 600 | 1.2 | 0.1 ± 0.1 |
| SZ Patient 18 | Male | 47 | 13 | 179 | 78 | 600 | 2.2 | 2.8 ± 0.4 |
| SZ Patient 19 | Male | 43 | 19 | 172 | 70 | 600 | 2.3 | 1.5 ± 0.2 |
| SZ Patient 20 | Male | 54 | 17 | 170 | 60 | 600 | 3.7 | 3.3 ± 0.6 |
| SZ Patient 21 | Male | 36 | 10 | 175 | 67 | 600 | 1.4 | 0.5 ± 0.4 |
| SZ Patient 22 | Male | 51 | 11 | 180 | 72 | 600 | 3.4 | 3.3 ± 0.6 |
| SZ Patient 23 | Male | 43 | 26 | 173 | 68 | 600 | 1.7 | 1.1 ± 0.4 |
| SZ Patient 24 | Male | 41 | 14 | 160 | 70 | 600 | 2.2 | 1.1 ± 0.5 |
| SZ Patient 25 | Male | 48 | 27 | 167 | 69 | 600 | 3.1 | 3.1 ± 0.2 |
| SZ Patient 26 | Male | 45 | 10 | 170 | 69 | 600 | 2.2 | 5.2 ± 0.1 |
| SZ Patient 27 | Male | 38 | 8 | 170 | 66 | 600 | 1.5 | 0.9 ± 0.3 |
| SZ Patient 28 | Male | 37 | 17 | 158 | 51 | 600 | 3.7 | 1.4 ± 0.4 |
| SZ Patient 29 | Male | 26 | 0.1 | 170 | 83 | 600 | 2.8 | 3.1 ± 0.1 |
| SZ Patient 30 | Female | 23 | 8 | 179 | 101 | 600 | 1.3 | 0.6 ± 0.1 |

Table S3. Detailed clinical information of HC volunteers involved in Li management.

| **Code** | **Sex** | **Age** | **Height/cm** | **Weight/kg** | **Daily dosage of Li_2_CO_3_/mg** | **Serum Li concentration**  **(μg mL^−1^)** | **δ^7^Li_serum-12h_/‰** |
| --- | --- | --- | --- | --- | --- | --- | --- |
| HC volunteer No.1 | Male | 25 | 172 | 62 | 600 | 3.4 | −3.0 ± 0.4 |
| HC volunteer No.2 | Male | 24 | 178 | 75 | 600 | 3.1 | −1.6 ± 0.4 |
| HC volunteer No.3 | Male | 24 | 168 | 75 | 600 | 2.6 | −2.3 ± 0.2 |
| HC volunteer No.4 | Male | 22 | 178 | 92 | 600 | 2.0 | −2.8 ± 0.4 |
| HC volunteer No.5 | Female | 29 | 168 | 65 | 600 | 2.6 | −6.4 ± 0.3 |
| HC volunteer No.6 | Female | 25 | 160 | 45 | 600 | 2.9 | −4.6 ± 0.3 |
| HC volunteer No.7 | Female | 24 | 165 | 51 | 600 | 3.8 | −4.4 ± 0.5 |
| HC volunteer No.8 | Female | 24 | 159 | 53.5 | 600 | 3.1 | −2.6 ± 0.3 |
| HC volunteer No.9 | Female | 23 | 158 | 52 | 600 | 3.2 | −1.2 ± 0.2 |
| HC volunteer No.10 | Male | 27 | 178 | 70 | 600 | 2.3 | −1.8 ± 0.5 |
| HC volunteer No.11 | Male | 23 | 172 | 83 | 600 | 2.8 | −3.6 ± 0.3 |
| HC volunteer No.12 | Female | 25 | 168 | 48 | 600 | 2.7 | −2.1 ± 0.3 |
| HC volunteer No.13 | Female | 23 | 160 | 56 | 600 | 3.1 | −2.2 ± 0.1 |
| HC volunteer No.14 | Female | 23 | 160 | 53 | 600 | 3.1 | −2.7 ± 0.2 |

**Reference:**

1. Mertens J, Wang QW, Kim Y, Yu DX, Pham S, Yang B, et al. Differential responses to lithium in hyperexcitable neurons from patients with bipolar disorder. Nature. 2015;527:95–9.

2. Hoecke KV, Belza J, Croymans T, Misra S, Claeys P, Vanhaecke F. Single-step chromatographic isolation of lithium from whole-rock carbonate and clay for isotopic analysis with multi-collector ICP-mass spectrometry. J Anal At Spectrom. 2015;30:2533–40.

3. Tian Y, Xiao Y, Chen YX, Sun H, Liu H, Tong F, et al. Serpentinite-derived low δ^7^Li fluids in continental subduction zones: constraints from the fluid metasomatic rocks (whiteschist) from the Dora-Maira Massif, Western Alps. Lithos. 2019;348–349:105177.

4. Pogge von Strandmann PAE, Vaks A, Bar-Matthews M, Ayalon A, Jacob E, Henderson GM. Lithium isotopes in speleothems: temperature-controlled variation in silicate weathering during glacial cycles. Earth Planet Sci Lett. 2017;469:64–74.

5. Thibon F, Weppe L, Montanes M, Telouk P, Vigier N. Lithium isotopic composition of reference materials of biological origin TORT-2, DORM-2, TORT-3, DORM-4, SRM-1400 and ERM-CE278k. J Anal At Spectrom. 2021;36:1381–8.

6. Balter V, Vigier N. Natural variations of lithium isotopes in a mammalian model. Metallomics. 2014;6:582–6.

7. Thibon F, Metian M, Oberhänsli F, Montanes M, Vassileva E, Orani AM, et al. Bioaccumulation of lithium isotopes in mussel soft tissues and implications for coastal environments. ACS Earth Space Chem*.* 2021;5:1407–17.

8. Thibon F, Weppe L, Churlaud C, Lacoue-Labarthe T, Gasparini S, Cherel Y, et al. Lithium isotopes in marine food webs: effect of ecological and environmental parameters. Front Environ Chem. 2023;3:29.

9. Veltri KL, Espiritu M, Singh G. Distinct genomic copy number in mitochondria of different mammalian organs. J Cell Physiol. 1990;143:160–4.

10. Larsen S, Nielsen J, Hansen CN, Nielsen LB, Wibrand F, Stride N, et al. Biomarkers of mitochondrial content in skeletal muscle of healthy young human subjects. J Physiol. 2012;590:3349–60.
